# Influence of a fatiguing exercise on lower limb electromyographic activities and co-contraction in overweight females during running

**DOI:** 10.1371/journal.pone.0322167

**Published:** 2025-05-06

**Authors:** AmirAli Jafarnezhadgero, Nastaran Moradzadeh, Ehsan Fakhri Mirzang, Heidar Sajedi, Sharon Dixon, Mohammad Akrami

**Affiliations:** 1 Department of Sport Biomechanics, Faculty of Educational Science and Psychology, University of Mohaghegh Ardabili, Ardabil, Iran; 2 Faculty of Health Science, Department Exercise and Sports Sciences for Disabled People, International Science and Technology University, Warsaw, Poland; 3 Department of Sport Sciences, Faculty of Public Health and Sport Sciences, University of Exeter, Exeter, United Kingdom; 4 Department of Engineering, Faculty of Environment, Science and Economy, University of Exeter, Exeter, United Kingdom; Ningbo University, CHINA

## Abstract

**Background:**

This study investigates the effects of a fatiguing exercise on lower limb electro-myographic activities and co-contraction in overweight females compared with normal weight females during running.

**Methods:**

Forty-eight females were divided into two groups. The first group included individuals with a normal body-mass-index. The second group comprised individuals classified as overweight/obese based on body-mass-index. Electromyography data from the tibialis anterior, gastrocnemius medialis, vastus lateralis, vastus medialis, rectus femoris, biceps femoris, and semitendinosus muscles were collected during running at constant speed using a surface electromyography system before and after a running induced fatigue.

**Findings:**

The results indicated significant main effects of the “Group” on tibialis anterior muscle activities during the loading phase (P = 0.040). Furthermore, the results showed significant main effects of “Fatigue” on rectus femoris (P = 0.028) and semitendinosus (P = 0.007) muscle activities during the loading phase. Paired-wise comparison demonstrated significantly greater rectus femoris and semitendinosus activities during the loading phase after the fatigue protocol. The results demonstrated significant main effects of “Fatigue” for general knee muscular co-contraction during early stance phase (P < 0.001). Paired-wise comparison demonstrated significantly greater general knee muscular co-contraction during early stance phase at post-test compared with pre-test. No significant main effect of “Group” and group-by-fatigue interactions were found for general and direct knee co-contraction during early stance phase (P > 0.05).

**Interpretation:**

Overall, our findings indicate that both fatigue and being overweight result in running pattern differences, but these occur through different mechanisms at a neuromuscular level. Neuromuscular responses to fatigue during running in overweight adults and in normal weight adults can be evaluated together, in order to optimize the modality of treatment and rehabilitation processes in overweight adults to reduce and/or prevent the risk of running related injury.

## 1. Introduction

Excessive body weight has been associated with an increased risk of running-related injuries [1,2]. For example, for novice runners enrolled in a running programme, 25% of overweight runners were reported to experience injuries during follow‐up, compared with 15% of participants with normal weight, where exposure to risk (running time per week) was similar for both groups [2]. This difference in injury risk may be a result of higher body mass resulting in larger magnitudes of repeated load being experienced by vulnerable structures. For example, greater structural loading has been highlighted as a potential mechanism for an observed greater incidence of chronic plantar heel pain/ plantar fasciitis in individuals with high body mass index (BMI) compared to those with normal body weight [3]. Whilst differences in injury may be contributed to by absolute differences in body mass, other factors associated with excessive body weight, such as increased fatigability, may also contribute to increased injury risk.

Fatigue has been highlighted as another potential risk factor for musculoskeletal running injuries [4]. Muscle response to running-induced fatigue includes an increase in mean absolute activation, suggested to reflect an increase in the recruitment and/or firing rate of motor units [5], increasing injury risk through mechanisms such as compromised movement control and proprioception [6]. Obesity is often marked by heightened fatigability and compromised motor performance, with adult obese individuals demonstrating elevated quadriceps fatigability during voluntary contractions, while exhibiting comparable levels of quadriceps fatigability in electrically elicited contractions when compared to adult lean subjects [7]. However, there is a paucity of research exploring the effects of a fatiguing exercise on lower limb electro-myographic activities in overweight females during running. It appears that running for prolonged durations increases muscle activation to control the moments at the joints of the lower extremity, potentially increasing both localized muscle fatigue and the compressive loading on those joints. Muscular co-contraction is a fundamental strategy employed by the human neuromuscular system to enhance joint stiffness, postural balance, and motion accuracy in dynamic conditions [8]. This physiological phenomenon constitutes one of the principal factors contributing to the enhancement of overall dynamic stability and the prevention of joint lesions. Furthermore, individuals utilize co-contraction in order to perform activities with demands that are higher relative to their capability [9]. In this context, muscular co-contraction would simplify the task by reducing the amount of reactive forces within the musculoskeletal system [10].

In a recent study, it has been reported that obesity increases muscle co-contraction of the soleus and tibialis anterior muscles around the ankle joint during static postural control [11]. Kim et al. [12] revealed that as weight increased, there was a corresponding increase in the rates of muscle activation, particularly in the tibialis anterior and soleus muscles. These findings suggest that heavier weight loads may increase activation of muscles that control ankle joints, potentially leading to muscle fatigue. This, in turn, was suggested to impair balance ability and increase the risk of falls. It has also been reported that obesity is associated with an excessive gastrocnemius medialis activity during the propulsive phase of walking and a high activity of soleus and tibialis anterior during the single support phase [13]. However, there has been no study that has investigated lower limb muscle co-contraction (especially in the knee joint) during running in overweight adults. Obtaining a better understanding of these factors could shed light on the underlying mechanisms that contribute to running alterations in overweight individuals. This information would be valuable to clinical practitioners as it could help to specify the appropriate type of physical conditioning.

Studies of running gait have identified distinct biomechanical characteristics between females and males [14,15] and thus a gender-specific approach is advocated. These differences include greater peak patellofemoral contact force loading rate [14], hip adduction excursion [14], knee abduction angular impulse [14] and angle [15], average vertical ground reaction force loading rate [14], knee internal rotation and ankle eversion [15], shorter step length [14], and lower hip flexion [15] being observed for females during running. In this regard, it is necessary to evaluate the running patterns of males and females separately.

This study investigates the influence of excessive body weight and running-related fatigue, on electromyographic (EMG) activities and the co-contraction of lower limb muscles during running, specifically to investigate whether the EMG response to fatigue differs for overweight females compared with those of normal weight. It was hypothesized that, (i) following a fatiguing run, runners would exhibit increased muscle activity of the lower limb muscles, and (ii) that the change in muscle activity would be greater for overweight female runners than for those of normal weight. It was also hypothesized (iii) that after fatigue, runners would demonstrate greater co-contraction of the muscles controlling knee flexion and providing the joint stiffness required to control knee flexion. It was also hypothesized (iv) that overweight female runners would demonstrate greater muscle activity and general co-contraction of the muscles as a compensatory mechanism in order to control joint movement.

## 2. Material and methods

The present study adopted a semi-experimental laboratory design. The sample consisted of female recreational runners exhibiting excessive body weight and normal weight. The World Masters Association age grading performance tables were used to classify runners’ performance levels. Recreational runners were classified as runners with an age-graded score of < 60% [16]. An age-graded score was computed via (www.howardgrubb.co.uk/athletics/ wmalookup06.html) according to the age, gender, recent race performance during the past 6 months (e.g., self-reported best time of 10 km, half-marathon or marathon race), and years of running practice [17]. Utilizing an available sampling approach, forty-eight females were categorized into two groups. The first group, denoted as N, comprised individuals with a normal body mass index (e.g., 20 ≤ BMI < 25 kg/m^2^). The second group, O, encompassed individuals categorized as overweight/obese (e.g., 35 ≥ BMI ≥ 25 kg/m^2^). Group characteristics are detailed in Table 1. Participants were recruited from the residents of Ardabil city through announcements on the World Wide Web and social media platforms. Exclusion criteria for all groups were as follows: a history of musculoskeletal surgery involving the trunk and/or lower limbs, outside of the BMI ranges, cardiorespiratory, neuromuscular, or orthopedic disorders; and lower limb length discrepancies exceeding 5 mm. Tape measure method was used in order to assess lower limb length discrepancies [18]. Subjects were positioned supine on a plinth. The examiner positioned the subject’s lower extremities in neutral hip rotation as determined by observation. The examiner then placed the subject’s medial malleoli together so that they met in a plane that approximated the mid-sagittal line of the body. The subject’s hip and knees were, therefore, in a position that closely approximated the anatomical position. The examiner held a blank tape measure between the thumb and the first finger of his hand nearest the subject’s pelvis. One end of the tape measure was placed on the anterior superior iliac spine at the site where the examiner believed he could palpate the origin of the sartorius muscle on the inferior portion of the anterior superior iliac spine. With the hand opposite to that holding the tape measure on the anterior superior iliac spine, the examiner gradually guided the tape down the antero-medial aspect of the subject’s thigh, patella, and lower leg until he made contact with the point where the subject’s medial malleolus sloped inferiorly and laterally. Another person recorded the value from the opposite surface of the tape. The examiner then repeated the same procedure on the subject’s opposite lower extremity. The same examiner then repeated the entire procedure to obtain a second pair of measurements. The leg-length difference was calculated by subtracting the right leg-length measurement from the left leg-length measurement [18]. According to the previous studies, leg-length difference of 5 mm or greater leads to biomechanical compensations in the spine and cause injuries to runners [19,20]*.* All participants exhibited heel striking patterns, as confirmed by kinetic data [21]. The age range of participants was between 18 and 35 years. The research protocol received approval from the ethics committee of the University of Mohaghegh Ardabili Iran (IR.UMA.REC.1402.011). Prior to the commencement of the study, participants provided written informed consent after being briefed on the benefits and potential risks associated with their participation. All sections of the present study have been performed in accordance with the Declaration of Helsinki. The data were accessed for research purposes at 2023-07-01. Also, the authors did not have access to information that could identify individual participants during or after data collection.

**Table 1:** Anthropometric characteristics in four groups.

Group	Normal weight	Over weight	P-value
Height (cm)	164.9 ± 4.5	160.6 ± 5.06	0.22
Mass (kg)	55.5 ± 7.30	79.2 ± 12.1	* < 0.001
Body mass index (kg/m^2^)	20.3 ± 2.0	30.6 ± 4.2	* < 0.001
Age (year)	21.9 ± 3.7	28.5 ± 7.21	0.03
Weekly mileage (km)	14.1 ± 1.7	14.0 ± 2.1	0.97

*Significant level P < 0.05

### Experimental protocol

The present study was conducted under two running conditions, namely, before and after fatigue induction. On the testing day, participants commenced with a four-minute session of dynamic stretching followed by a five-minute warm-up involving light jogging (rated between 10 to 11 on a 6–20 Borg scale). Subsequently, both before and after the fatigue protocol, participants engaged in running activities while wearing consistent running shoes, maintaining a steady speed of approximately 3.3 m/s (with a 5% variability) along an 18-meter overground straight walkway featuring an embedded force plate. The average running speed was calculated by dividing the running distance (18 m) by the recorded running time, assessed using a chronometer. The participants were instructed to perform each of the running trials along an 18-meter overground straight walkway at 5.45 s (with a 5% variability). Each participant performed 6 successful running trials before and after a fatigue protocol, where a successful trial was achieved when participants performed an optimal landing of their dominant foot at the center of the force plate (Bertec Corporation, Columbus, 4060–07 Model, OH, United States) sampled at 1000 Hz [22]. After fatigue induction, the biomechanical testing was conducted immediately. Running stance phase was defined as the interval from ground contact (vertical ground reaction force >10 N) to toe of (vertical ground reaction force <10 N). Kinetic data were filtered using a third-order low-pass Butterworth filter with a cutoff frequency of 50 Hz.

### Electromyography recordings

Electromyographic patterns were recorded during the running trial using bipolar Ag/AgCl surface electrodes positioned parallel to the muscle fiber direction, maintaining an inter-electrode distance of 20 mm. Rigorous skin preparation was executed to ensure skin impedance remained at or below 5000Ω. The EMG data were meticulously recorded at a frequency of 1000 Hz through an EMG system (Data LITE EMG, Biometrics Ltd, England). Data acquisition focused on eight prominent surface muscles of the lower extremity, including the tibialis anterior (TA), gastrocnemius medialis (Gas-M), vastus lateralis (VL), vastus medialis (VM), rectus femoris (RF), biceps femoris (BF), and semitendinosus (ST) muscles, aligning with the recommendations put forth by SENIAM [23]. EMG signals underwent sampling at a conversion rate of 1000 Hz with 16-bit resolution (amplitude range ± 5V; band-pass filtered, 10–500 Hz; input impedance > 10 Ohm; common mode-rejection ratio >110 dB), facilitated by a portable Wi-Fi transmission device. The run was partitioned into two distinct phases for EMG data analysis: the initial 0‒50% stance phase (‘early stance phase’) and the subsequent 50‒100% stance phase (late stance phase’) [24,25]. Root mean square (RMS) values during each phase were calculated. Employing a handheld dynamometer, maximum voluntary isometric contraction (MVIC) was evaluated for each recorded muscle to standardize RMS values during running to the peak RMS values during maximal voluntary activation level [26]. Appendix 1 describes the muscle-specific MVIC tests [27]. All normalization procedures were realized in accordance with recommendations from Besomi et al. (2020) [28]. For example, the participants were encouraged to perform the tests at maximal effort [28]. Three test trials were conducted with 1–2 min rest periods inbetween tests [28]. For measuring MVIC, an isometric belt (where the joint is locked) was used (set for zero velocity) [28]. This instrument is important to control testing factors that can influence the output and facilitate the production of maximal contraction. The maximum value of the MVIC test was considered for normalization purposes [28].

The total activation of the agonist and antagonist muscles was calculated as general co-contraction [29]. Directed co-contraction ratios (DCCR) were calculated for the medial (semitendinosus and vastus medialis)/lateral (biceps femoris and vastus lateralis) muscles (MLDCCR), and the knee flexors (semitendinosus, biceps femoris, and gluteus medius)/extensors (vastus lateralis, vastus medialis, and rectus femoris) (FEDCCR) [29]. The DCCRs were calculated as follows [29]:

If agonist mean EMG > antagonist mean EMG;

DCCR = 1 - antagonist mean EMG/ agonist mean EMG

Else

DCCR = agonist mean EMG/ antagonist mean EMG – 1

In these equations if agonists (e.g., extensors or medial muscles) were more active than the antagonists (e.g., extensors or lateral muscles) the DCCR would be above zero, and vice versa. Maximum co-contraction would be represented with a DCCR equal to zero, while a minimum co-contraction is indicated with a DCCR of 1 or -1.

### Fatigue protocol

The fatigue running protocol was executed on a motorized treadmill at zero gradient (Horizon Fitness, Omega GT, USA) [30,31]. Participants initiated the test with a walking speed of 6 km/h, followed by a subsequent increment of 1 km/h every 2 minutes [30,31]. The assessment of perceived exertion, as indicated by the 6–20 Borg scale, was obtained from participants at the conclusion of each stage [32]. Once participants reported a perceived exertion of 13 or greater, the treadmill velocity was set to facilitate a phase of steady-state running. Throughout this steady-state running segment, perceived exertion ratings were evaluated at 30-second intervals. The fatiguing protocol was concluded if either two minutes of steady-state running were sustained with a perceived exertion surpassing 17 on the 6–20 Borg scale or achieving ≥80% of the maximum heart rate [33]. Following the fatigue protocol, the post-test was conducted in a manner consistent with the pre-test.

### Statistical analysis

The running variables extracted were averaged over six trials for each participant. Presentation of data is in the form of group mean values and standard deviations. The normal distribution of the data was verified through the Shapiro-Wilk test. Custom scripts (MATLAB R2022a, The MathWorks, Natick, USA) were employed for all analyses. Main effects of body mass (normal weight, overweight) were computed utilizing a two-way ANOVA with repeated measures for each dependent variable. Effect sizes were assessed employing partial ETA squared (0.01 < ETA ≤ 0.06: small effect size (ES); 0.06 > ETA < 0.14 = moderate effect size; ETA ≥ 0.14: high effect size). The significance level was predetermined at p < 0.05. The p-value for the post-hoc analysis was adjusted using Bonferroni correction. The Bonferroni method was used to control family-wise Type I error rate to be P ≤ 0.05 for multiple comparisons (Bonferroni significance level P ≤ 0.025). All analyses were executed using SPSS 23.

## 3. Results

Anthropometric characteristics of the two groups are presented in Table 1, highlighting the distinct groups in terms of body mass and body mass index.

The results demonstrated significant main effects of “Fatigue” for RF (P = 0.024, ƞ^2^ = 0.103) and ST (P = 0.010, ƞ^2^ = 0.137) muscles during early stance phase (Table 2). Paired-wise comparison demonstrated significantly greater RF and ST muscle activities during early stance phase at the post-test compared with the pre-test. The results demonstrated significant main effects of “Group” for TA muscle activities during early stance phase (P = 0.009, ƞ^2^ = 0.139). Paired-wise comparison demonstrated significantly greater TA muscle activities in the overweight group than that normal weight group. No significant group-by-fatigue interactions were found for muscle activity during early stance phase (P > 0.05) (Table 2).

**Table 2:** Mean and standard deviation of the normalised electromyography amplitude (%MVIC) of lower limb muscles during early stance phase.

Muscles	Normal weight		Overweight		Fatigue Sig. (Eta Square)	Group Sig. (Eta Square)	Fatigue-by-group Interactions Sig. (Eta Square)
	Pre-test	Post-test	Pre-test	Post-test			
TA	55.7 ± 20.5	57.7 ± 23.0	66.1 ± 29.0	89.1 ± 61.3	0.103(0.057)	**0.009(0.139)**	0.171(0.40)
Gas-M	22.8 ± 16.8	21.1 ± 18.6	22.9 ± 14.6	24.9 ± 21.5	0.958(0.000)	0.605(0.006)	0.609(0.006)
VL	59.8 ± 50.5	77.1 ± 53.9	61.8 ± 53.9	76.7 ± 73.9	0.055(0.078)	0.959(0.000)	0.889(0.000)
VM	48.2 ± 35.2	52.2 ± 33.8	37.3 ± 16.1	64.3 ± 58.2	0.055(0.078)	0.941(0.000)	0.152(0.044)
RF	29.5 ± 14.0	48.2 ± 74.7	35.8 ± 20.0	57.6 ± 44.4	**0.026(0.103)**	0.421(0.014)	0.856(0.001)
BF	29.2 ± 10.2	41.4 ± 41.1	44.3 ± 28.8	48.5 ± 24.3	0.076(0.067)	0.111(0.054)	0.378(0.017)
ST	38.8 ± 27.9	57.1 ± 46.5	44.9 ± 27.5	73.7 ± 57.4	**0.010(0.137)**	0.183(0.038)	0.546(0.008)

**Notes**: TA, Tibialis anterior; Gas-M, Gastrocnemius medialis; VL, Vastus lateralis; VM, Vastus medialis; RF, Rectus femoris; BF, Biceps femoris; ST, Semitendinosus.

The results demonstrated significant main effects of “Fatigue” for VM (P < 0.001, ƞ^2^ = 0.534), RF (P = 0.002, ƞ^2^ = 0.197) and ST (P = 0.011, ƞ^2^ = 0.133) muscle activities during late stance phase (Table 3). Paired-wise comparison demonstrated significantly greater VM, RF and ST muscles activities during late stance phase at post-test compared with the pre-test during push off phase. The results demonstrated significant main effects of “Group” for TA muscle activities (P = 0.045, ƞ^2^ = 0.087) and RF (P = 0.024, ƞ^2^ = 0.103) during late stance phase. Paired-wise comparison demonstrated significantly greater TA and RF muscle activities in the overweight group than that normal weigh group during late stance phase. Also the results demonstrated no significant group-by-fatigue interactions for muscle activities during late stance phase (P > 0.05).

**Table 3:** Mean and standard deviation of the normalised electromyography amplitude (%MVIC) of lower limb muscles during late stance phase.

Muscles	Normal weight		Overweight		Fatigue Sig. (Eta Square)	Group Sig. (Eta Square)	Group-by-Fatigue Interactions Sig. (Eta Square)
	Pre-test	Post-test	Pre-test	Post-test			
TA	46.3 ± 17.1	50.3 ± 20.7	49.9 ± 21.8	67.8 ± 41.5	0.081(0.066)	**0.045(0.087)**	0.195(0.037)
Gas-M	145.9 ± 109.6	132.3 ± 70.8	142.4 ± 68.2	152.5 ± 62.3	0.912(0.005)	0.624(0.005)	0.459(0.012)
VL	94.0 ± 75.3	95.5 ± 56.2	68.2 ± 27.0	122.7 ± 119.5	0.074(0.068)	0.967(0.000)	0.090(0.061)
VM	48.2 ± 35.2	87.0 ± 33.5	37.3 ± 16.1	89.9 ± 44.5	**<.001(0.534)**	0.598(0.006)	0.281(0.025)
RF	44.8 ± 30.3	56.0 ± 40.9	71.7 ± 44.6	86.8 ± 60.4	**0.002(0.197)**	**0.026(0.103)**	0.627(0.005)
BF	37.6 ± 17.4	52.7 ± 42.8	47.7 ± 29.0	48.4 ± 21.3	0.113(0.054)	0.679(0.004)	0.145(0.046)
ST	42.6 ± 19.0	70.9 ± 58.4	53.5 ± 28.5	69.5 ± 48.6	**0.011(0.133)**	0.584(0.007)	0.469(0.011)

**Notes**: TA, Tibialis anterior; Gas-M, Gastrocnemius medialis; VL, Vastus lateralis; VM, Vastus medialis; RF, Rectus femoris; BF, Biceps femoris; ST, Semitendinosus.

The results demonstrated significant main effects of “Fatigue” for general knee muscular co-contraction during early stance phase (P < 0.001, ƞ^2^ = 0.259) (Table 4). Paired-wise comparison demonstrated significantly greater general knee muscular co-contraction during early stance phase at post-test compared with pre-test. No significant main effect of “Group” and group-by-fatigue interactions were found for general and direct knee co-contraction during early stance phase (P > 0.05) (Table 4).

**Table 4:** Mean and standard deviation of the knee muscular co-contraction of lower limb muscles during early stance phase.

Muscles	Normal weight		Overweight		Fatigue Sig (Eta Square)	Group Sig (Eta Square)	Group-by-Fatigue Interactions Sig. (Eta Square)
	Pre-test	Post-test	Pre-test	Post-test			
General knee co-contraction	205.8 ± 76.7	276.1 ± 139.4	224.2 ± 106.4	321.0 ± 171.3	<0.001* (0.244)	0.301(0.023)	0.545(0.008)
Directed knee Flexor/Extensor co-contraction	0.3 ± 0.4	0.0 ± 0.8	0.2 ± 0.5	0.8 ± 0.5	0.300 (0.99)	0.727 (0.003)	0.274 (0.026)
Directed knee medio/lateral co-contraction	-0.2 ± 1.0	-0.1 ± 0.7	0.7 ± 0.4	-0.3 ± 0.7	0.183 (0.038)	0.747 (0.002)	0.053 (0.079)

The results demonstrated significant main effects of “Fatigue” for general knee muscular co-contraction during late stance phase (P < 0.001, ƞ^2^ = 0.400) (Table 5). Paired-wise comparison demonstrated significantly greater general knee co-contraction during late stance phase at post-test compared with the pre-test (P < 0.001, d = 0.92). Also, results demonstrated significant main effect of “Fatigue” for directed knee medio/lateral co-contraction (P=<0.001, ƞ^2^ = 0.241). Paired-wise comparison demonstrated significantly lower directed knee medio/lateral co-contraction during late stance phase at post-test compared with the pre-test (P < 0.001, d = 0.97) (Table 5). No significant main effect of “Group” and group-by-fatigue interactions were found for general and directed knee co-contraction during late stance phase (P > 0.05) (Table 5).

**Table 5:** Mean and standard deviation of the co-contraction of lower limb muscles during late stance phase.

Muscles	Normal weight		Overweight		Fatigue Sig (Eta)	Group Sig (Eta)	Group-by-Fatigue Interactions Sig. (Eta Square)
	Pre-test	Post-test	Pre-test	Post-test			
General knee co-contraction	267.4 ± 98.2	362.3 ± 129.8	278.6 ± 91.9	417.5 ± 186.5	<0.001* (0.400)	0.298(0.023)	0.304(0.023)
Directed knee Flexor/Extensor co-contraction	0.4 ± 0.3	0.3 ± 0.7	0.3 ± 0.4	0.5 ± 0.2	0.649 (0.005)	0.614 (0.006)	0.126 (0.050)
Directed knee medio/lateral co-contraction	0.1 ± 0.4	-0.2 ± 0.5	0.1 ± 0.2	-0.2 ± 0.8	<0.001* (0.241)	0.881 (0.000)	0.896 (0.000)

## 4. Discussion

This study aimed to assess the impact of excessive body weight and running related fatigue and their interactions on electromyography activities and co-contraction of lower limb muscles during running, and to investigate whether the response to fatigue differs for overweight versus normal weight runners. By dividing ground contact into early and late stance phases, it has been possible to interpret observations in relation to the function of the respective muscle groups, both in terms of the effect of excessive body weight and the influence of a fatiguing exercise.

Our first hypothesis (i) that, following a fatiguing run, runners would exhibit increased muscle activity of the lower limb muscles has been supported just for RF muscle, with greater RF activity during the early stance phase post-activity. The RF contributes eccentrically to control of knee flexion during this cushioning phase of stance, likely contributing to the observation of greater activity following a fatiguing run. Moreover, our first hypothesis that, following a fatiguing run, runners would exhibit increased muscle activity of the lower limb muscles has been supported for quadriceps (RF and VM) and hamstring (ST) muscles during late stance phase. The observation that, during the late stance phase, muscles of both the quadriceps (RF and VM) and hamstring (ST) groups demonstrated greater activity post-run, supports the additional role of the hamstrings during this propulsive phase of stance [34]. The greater muscle activity during the early and late stance phases after the fatiguing protocol may have implications for injury, providing a possible mechanism regarding the suggested contribution of fatigue to the incidence of musculoskeletal injuries occurring in sports training and competition [35]. The EMG amplitude values are related with muscle activation and are altered by the level of firing rate and motor unit recruitment [5]. EMG amplitude values increment during constant, sustained workloads or force levels as the result of fatigue [36–38] or with increases in training intensity to accommodate the greater workload [39–41]. Moreover, the RF muscle is biarticular and its role in both hip flexion and leg extension during running along with its fiber type characteristics explain its greater fatigability [42,43] during running. It has been reported that ~74% of the RF volume was activated during running, while only 53% of the monoarticular vastus muscle group (VL, VM and vastus intermedius) contributed to the force generation [44]. The RF muscle is characterized by a higher percentage of fast-twitch glycolytic fibers relative to the vastus muscle group [45,46], which rely heavily on anaerobic energy generation and would contribute to greater metabolic by product accumulation [47]. Therefore, the special muscle responses and higher fatigability of the RF to running induced fatigue could possibility have been contributed to by differences in muscle architecture (bi- versus mono-articular) or fiber type between the RF and vastus muscles. Exploring the interaction between body weight and fatigue in running advances the understanding of running injury prevention or performance optimization. Based on our results, overweight participants do not experience greater increases in muscle activity after fatigue compared to normal-weight participants, and thus hypothesis (ii) is not supported. The division of stance into two phases has allowed interpretation of the functional impact of our results. In future studies, it may be possible to provide a more detailed analysis and reveal additional functional implications using methods such as machine learning [48,49].

The observation of greater general knee co-contraction during the early and late stance phases following the fatiguing run supports study hypothesis (iii) regarding co-contraction. This increased co-contraction reflects the greater activity of the muscles controlling knee flexion and extension, and provides the joint stiffness required to resist excessive knee flexion. This greater general knee muscular co-contraction during early and late stance phases after the fatiguing protocol suggests a potential for increased injury risk with increased levels of fatigue. The additional observation of lower directed knee medio/lateral co-contraction during late stance phase after fatigue protocol compared with before it may suggest fatigue has a role in osteoarthritis (OA) development and progression. The change from a positive to negative magnitude of directed knee medio/lateral co-contraction highlights a change in dominance from medial to lateral muscles following fatigue. Since a bias of muscle activation to the lateral muscles acts to generate an internal abduction moment at the knee, this potentially contributes to a reduction in medial knee loading, where high medial loading has been associated with OA risk and progression. This suggests an adjustment in muscle co-contraction following fatigue which acts to reduce OA risk [50–52]. However, more detailed study, for example to include measurement of knee kinetics, is required to investigate this interpretation further.

Hypothesis (iv) that overweight runners would demonstrate greater muscle activities and general co-contraction of the muscles as a compensatory mechanism in order to control joint movement has not been supported. Results demonstrated greater TA muscle activities during running early and late stance phase and greater RF activity during late stance phase in the overweight group than that normal weight group. During the early stance phase, the plantar-flexion movement of the foot is controlled with eccentric contraction of TA, with this movement also associated with provision of shock absorption [53,54]. TA is also activated during the early stance phase in order to oppose the passive pronation motion that occurs at heel contact [55]. The greater TA activity during the early stance phase in the overweight group may be a mechanism adopted in these individuals in order to provide the required shock absorption to manage the potential high loads associated with greater body weight. This suggestion is supported by previous observations that, when presented relative to bodyweight, ground reaction force during the early stance phase tends to be lower for overweight runners than observed for normal weight runners [55]. It is suggested that the greater TA muscle activity for the overweight group may contribute to the greater incidence of injuries such as exercise induced lower limb pain [11] due to the greater demand placed on this muscle group during running. Kim et al. [11] revealed that as weight increased, there was a corresponding increase in the rates of muscle activation, particularly in the TA and soleus muscles. These findings suggest that heavier weight loads may increase activation of muscles that control ankle joints, potentially leading to muscle fatigue [13]. This, in turn, can impair balance ability and increase the risk of falls. In another study, it has been reported that obesity was associated with an excessive Gas-M activity during the propulsive phase of walking and a high activity of soleus and TA during the single support phase [13]. Excessive muscle activity reduces the degrees of freedom in the postural control system [56] and might inhibit smooth joint motion and restrict dynamic performance in walking and running [57,58]. These adaptive neuromuscular responses could be explained by the biomechanical modifications of running related to the mobilization of excessive body mass. Indeed, DeVita and Hortobágyi (2003) reported that joint torques and powers were 88% and 61% higher respectively in obese individuals compared to non-obese, suggesting that obese adults reorganize their neuromuscular function in response to the excessive body mass to mobilize during walking and running [59]. The observed adaptive neuromuscular strategies may be taken as a self-optimization by which overweight adults can operate within their strength and functional capacities to manage the propulsion of the body and increase stability. However, higher muscle activity is associated with higher energy cost [60,61], meaning that this adaptation is likely to result in earlier fatigue. Moreover, our results did not show significant changes in general and directed knee co-contraction of the muscles in overweight participants compared to normal-weight participants.

The observation of no significant group-by-fatigue interaction for muscle activity or co-contraction during the early stance phase suggests that the greater injury incidence reported for overweight compared with normal weight runners is not contributed to by different levels of muscular fatigue. Regardless of the bodyweight, runners appear to respond to the same extent, both in terms of increased absolute muscle activity and co-contraction. This response for running is in contrast to previous reports of overweight participants exhibiting heightened fatigability and compromised motor performance in response to a fatiguing protocol [62], suggesting that the response to running differs to response to repeated voluntary contractions in a seated task, highlighting the importance of understanding specific functional tasks.

The focus of this study on muscle activity and co-contraction provides novel evidence regarding the increase in these variables following a fatiguing run, with no evidence that carrying excessive body weight enhances these effects. The interpretation of these observations would be enhanced by the addition of kinematic data, for example allowing suggestions regarding the subsequent effect on joint movement to be explored. Further studies simultaneously collecting kinematic data are therefore recommended. It is acknowledged that the inclusion of only female participants limits the application of these results to the wider population, and thus our findings cannot be generalized to include the male running population. This focus on female runners provides a heterogeneous population, strengthening the internal validity of the study. However, in order to understand the wider running population, it is suggested that further study should include male runners.

## 5. Conclusions

The results showing increased muscle activities and co-contraction after the fatigue protocol compared to before it may have implications for increased energy cost when running in a fatigued state. The absence of interactions between fatigue and bodyweight group on lower limb muscle activities and knee muscular co-contraction during running suggests that the difference in reported injury incidence between these groups is not contributed to by different responses to fatigue. The finding of higher tibialis anterior muscle activities in the overweight group compared to the normal weight group during the loading phase highlight a possible mechanism for greater injury in overweight versus normal weight runners. These results suggest that interventions to reduce injury risk in overweight runners should focus on improving strength and function of the TA muscle group. In addition, interventions such as functional conditioning should be explored, to reduce fatigue of the muscle groups crossing the knee joint, aiming to delay the observed increase in muscle activity and co-contraction. Overall, our findings indicate that both fatigue and being overweight result in running pattern differences, but these occur through different mechanisms at a neuromuscular level. Electromyography analyses of lower limb muscle activities indicate that the interaction of the influence of fatigue and being overweight during running is different from those related to the individual effects of fatigue and overweight. Therefore, neuromuscular responses to fatigue during running in overweight adults and in normal weight adults can be evaluated together, in order to optimize the modality of treatment and rehabilitation processes in overweight adults to reduce and/or prevent the risk of running related injury.

## Supporting information

Appendix 1 Tests for the assessment of maximum voluntary isometric contraction. (DOCX)

S1 data Data. (DOCX)
