# Supplementary material for: Influence of a fatiguing exercise on lower limb electromyographic activities and co-contraction in overweight females during running
Source: PLoS One. 2025 May 6;20(5):e0322167. doi: 10.1371/journal.pone.0322167 (PMC12054892; doi:10.1371/journal.pone.0322167)
Supplement: S1 data — (DOCX) [file pone.0322167.s002.docx]

**TA - early stance**

| **Within-Subjects Factors** | |
| --- | --- |
| Measure: MEASURE_1 | |
| fatige | Dependent Variable |
| 1 | nTAlrPreA |
| 2 | nTAlrPosA |

| **Between-Subjects Factors** | | | |
| --- | --- | --- | --- |
|  | | Value Label | N |
| Groups | 1.00 | noramweieght | 24 |
|  | 2.00 | over weight | 24 |

| **Descriptive Statistics** | | | | |
| --- | --- | --- | --- | --- |
|  | Groups | Mean | Std. Deviation | N |
| nTAlrPreA | noramweieght | 55.7153 | 20.56368 | 24 |
|  | over weight | 66.1502 | 29.04973 | 24 |
|  | Total | 60.9327 | 25.44997 | 48 |
| nTAlrPosA | noramweieght | 57.7770 | 23.09503 | 24 |
|  | over weight | 89.1864 | 61.36947 | 24 |
|  | Total | 73.4817 | 48.53802 | 48 |

| **Multivariate Tests^a^** | | | | | | | | | |
| --- | --- | --- | --- | --- | --- | --- | --- | --- | --- |
| Effect | | Value | F | Hypothesis df | Error df | Sig. | Partial Eta Squared | Noncent. Parameter | Observed Power^c^ |
| fatige | Pillai's Trace | .057 | 2.770^b^ | 1.000 | 46.000 | .103 | .057 | 2.770 | .371 |
|  | Wilks' Lambda | .943 | 2.770^b^ | 1.000 | 46.000 | .103 | .057 | 2.770 | .371 |
|  | Hotelling's Trace | .060 | 2.770^b^ | 1.000 | 46.000 | .103 | .057 | 2.770 | .371 |
|  | Roy's Largest Root | .060 | 2.770^b^ | 1.000 | 46.000 | .103 | .057 | 2.770 | .371 |
| fatige * Groups | Pillai's Trace | .040 | 1.935^b^ | 1.000 | 46.000 | .171 | .040 | 1.935 | .275 |
|  | Wilks' Lambda | .960 | 1.935^b^ | 1.000 | 46.000 | .171 | .040 | 1.935 | .275 |
|  | Hotelling's Trace | .042 | 1.935^b^ | 1.000 | 46.000 | .171 | .040 | 1.935 | .275 |
|  | Roy's Largest Root | .042 | 1.935^b^ | 1.000 | 46.000 | .171 | .040 | 1.935 | .275 |
| a. Design: Intercept + Groups  Within Subjects Design: fatige | | | | | | | | | |
| b. Exact statistic | | | | | | | | | |
| c. Computed using alpha = .05 | | | | | | | | | |

| **Tests of Within-Subjects Effects** | | | | | | | | | |
| --- | --- | --- | --- | --- | --- | --- | --- | --- | --- |
| Measure: MEASURE_1 | | | | | | | | | |
| Source | | Type III Sum of Squares | df | Mean Square | F | Sig. | Partial Eta Squared | Noncent. Parameter | Observed Power^a^ |
| fatige | Sphericity Assumed | 3779.424 | 1 | 3779.424 | 2.770 | .103 | .057 | 2.770 | .371 |
|  | Greenhouse-Geisser | 3779.424 | 1.000 | 3779.424 | 2.770 | .103 | .057 | 2.770 | .371 |
|  | Huynh-Feldt | 3779.424 | 1.000 | 3779.424 | 2.770 | .103 | .057 | 2.770 | .371 |
|  | Lower-bound | 3779.424 | 1.000 | 3779.424 | 2.770 | .103 | .057 | 2.770 | .371 |
| fatige * Groups | Sphericity Assumed | 2639.550 | 1 | 2639.550 | 1.935 | .171 | .040 | 1.935 | .275 |
|  | Greenhouse-Geisser | 2639.550 | 1.000 | 2639.550 | 1.935 | .171 | .040 | 1.935 | .275 |
|  | Huynh-Feldt | 2639.550 | 1.000 | 2639.550 | 1.935 | .171 | .040 | 1.935 | .275 |
|  | Lower-bound | 2639.550 | 1.000 | 2639.550 | 1.935 | .171 | .040 | 1.935 | .275 |
| Error(fatige) | Sphericity Assumed | 62759.236 | 46 | 1364.331 |  |  |  |  |  |
|  | Greenhouse-Geisser | 62759.236 | 46.000 | 1364.331 |  |  |  |  |  |
|  | Huynh-Feldt | 62759.236 | 46.000 | 1364.331 |  |  |  |  |  |
|  | Lower-bound | 62759.236 | 46.000 | 1364.331 |  |  |  |  |  |
| a. Computed using alpha = .05 | | | | | | | | | |

| **Tests of Within-Subjects Contrasts** | | | | | | | | | |
| --- | --- | --- | --- | --- | --- | --- | --- | --- | --- |
| Measure: MEASURE_1 | | | | | | | | | |
| Source | fatige | Type III Sum of Squares | df | Mean Square | F | Sig. | Partial Eta Squared | Noncent. Parameter | Observed Power^a^ |
| fatige | Linear | 3779.424 | 1 | 3779.424 | 2.770 | .103 | .057 | 2.770 | .371 |
| fatige * Groups | Linear | 2639.550 | 1 | 2639.550 | 1.935 | .171 | .040 | 1.935 | .275 |
| Error(fatige) | Linear | 62759.236 | 46 | 1364.331 |  |  |  |  |  |
| a. Computed using alpha = .05 | | | | | | | | | |

| **Tests of Between-Subjects Effects** | | | | | | | | |
| --- | --- | --- | --- | --- | --- | --- | --- | --- |
| Measure: MEASURE_1 | | | | | | | | |
| Transformed Variable: Average | | | | | | | | |
| Source | Type III Sum of Squares | df | Mean Square | F | Sig. | Partial Eta Squared | Noncent. Parameter | Observed Power^a^ |
| Intercept | 433613.804 | 1 | 433613.804 | 305.611 | .000 | .869 | 305.611 | 1.000 |
| Groups | 10505.652 | 1 | 10505.652 | 7.404 | .009 | .139 | 7.404 | .759 |
| Error | 65266.677 | 46 | 1418.841 |  |  |  |  |  |
| a. Computed using alpha = .05 | | | | | | | | |

| **Parameter Estimates** | | | | | | | | | | |
| --- | --- | --- | --- | --- | --- | --- | --- | --- | --- | --- |
| Dependent Variable | Parameter | B | Std. Error | t | Sig. | 95% Confidence Interval | | Partial Eta Squared | Noncent. Parameter | Observed Power^b^ |
|  |  |  |  |  |  | Lower Bound | Upper Bound |  |  |  |
| nTAlrPreA | Intercept | 66.150 | 5.137 | 12.877 | .000 | 55.810 | 76.491 | .783 | 12.877 | 1.000 |
|  | [Groups=1.00] | -10.435 | 7.265 | -1.436 | .158 | -25.059 | 4.189 | .043 | 1.436 | .290 |
|  | [Groups=2.00] | 0^a^ | . | . | . | . | . | . | . | . |
| nTAlrPosA | Intercept | 89.186 | 9.464 | 9.423 | .000 | 70.136 | 108.237 | .659 | 9.423 | 1.000 |
|  | [Groups=1.00] | -31.409 | 13.385 | -2.347 | .023 | -58.351 | -4.467 | .107 | 2.347 | .632 |
|  | [Groups=2.00] | 0^a^ | . | . | . | . | . | . | . | . |
| a. This parameter is set to zero because it is redundant. | | | | | | | | | | |
| b. Computed using alpha = .05 | | | | | | | | | | |

Gas-M -Early stance phase

**General Linear Model**

| **Within-Subjects Factors** | |
| --- | --- |
| Measure: MEASURE_1 | |
| fatige | Dependent Variable |
| 1 | nGClrPreA |
| 2 | nGClrPosA |

| **Between-Subjects Factors** | | | |
| --- | --- | --- | --- |
|  | | Value Label | N |
| Groups | 1.00 | noramweieght | 24 |
|  | 2.00 | over weight | 24 |

| **Descriptive Statistics** | | | | |
| --- | --- | --- | --- | --- |
|  | Groups | Mean | Std. Deviation | N |
| nGClrPreA | noramweieght | 22.8377 | 16.80995 | 24 |
|  | over weight | 22.9412 | 14.67153 | 24 |
|  | Total | 22.8895 | 15.60836 | 48 |
| nGClrPosA | noramweieght | 21.1658 | 18.61204 | 24 |
|  | over weight | 24.9930 | 21.56353 | 24 |
|  | Total | 23.0794 | 20.02011 | 48 |

| **Multivariate Tests^a^** | | | | | | | | | |
| --- | --- | --- | --- | --- | --- | --- | --- | --- | --- |
| Effect | | Value | F | Hypothesis df | Error df | Sig. | Partial Eta Squared | Noncent. Parameter | Observed Power^c^ |
| fatige | Pillai's Trace | .000 | .003^b^ | 1.000 | 46.000 | .958 | .000 | .003 | .050 |
|  | Wilks' Lambda | 1.000 | .003^b^ | 1.000 | 46.000 | .958 | .000 | .003 | .050 |
|  | Hotelling's Trace | .000 | .003^b^ | 1.000 | 46.000 | .958 | .000 | .003 | .050 |
|  | Roy's Largest Root | .000 | .003^b^ | 1.000 | 46.000 | .958 | .000 | .003 | .050 |
| fatige * Groups | Pillai's Trace | .006 | .265^b^ | 1.000 | 46.000 | .609 | .006 | .265 | .080 |
|  | Wilks' Lambda | .994 | .265^b^ | 1.000 | 46.000 | .609 | .006 | .265 | .080 |
|  | Hotelling's Trace | .006 | .265^b^ | 1.000 | 46.000 | .609 | .006 | .265 | .080 |
|  | Roy's Largest Root | .006 | .265^b^ | 1.000 | 46.000 | .609 | .006 | .265 | .080 |
| a. Design: Intercept + Groups  Within Subjects Design: fatige | | | | | | | | | |
| b. Exact statistic | | | | | | | | | |
| c. Computed using alpha = .05 | | | | | | | | | |

| **Tests of Within-Subjects Effects** | | | | | | | | | |
| --- | --- | --- | --- | --- | --- | --- | --- | --- | --- |
| Measure: MEASURE_1 | | | | | | | | | |
| Source | | Type III Sum of Squares | df | Mean Square | F | Sig. | Partial Eta Squared | Noncent. Parameter | Observed Power^a^ |
| fatige | Sphericity Assumed | .866 | 1 | .866 | .003 | .958 | .000 | .003 | .050 |
|  | Greenhouse-Geisser | .866 | 1.000 | .866 | .003 | .958 | .000 | .003 | .050 |
|  | Huynh-Feldt | .866 | 1.000 | .866 | .003 | .958 | .000 | .003 | .050 |
|  | Lower-bound | .866 | 1.000 | .866 | .003 | .958 | .000 | .003 | .050 |
| fatige * Groups | Sphericity Assumed | 83.194 | 1 | 83.194 | .265 | .609 | .006 | .265 | .080 |
|  | Greenhouse-Geisser | 83.194 | 1.000 | 83.194 | .265 | .609 | .006 | .265 | .080 |
|  | Huynh-Feldt | 83.194 | 1.000 | 83.194 | .265 | .609 | .006 | .265 | .080 |
|  | Lower-bound | 83.194 | 1.000 | 83.194 | .265 | .609 | .006 | .265 | .080 |
| Error(fatige) | Sphericity Assumed | 14420.124 | 46 | 313.481 |  |  |  |  |  |
|  | Greenhouse-Geisser | 14420.124 | 46.000 | 313.481 |  |  |  |  |  |
|  | Huynh-Feldt | 14420.124 | 46.000 | 313.481 |  |  |  |  |  |
|  | Lower-bound | 14420.124 | 46.000 | 313.481 |  |  |  |  |  |
| a. Computed using alpha = .05 | | | | | | | | | |

| **Tests of Within-Subjects Contrasts** | | | | | | | | | |
| --- | --- | --- | --- | --- | --- | --- | --- | --- | --- |
| Measure: MEASURE_1 | | | | | | | | | |
| Source | fatige | Type III Sum of Squares | df | Mean Square | F | Sig. | Partial Eta Squared | Noncent. Parameter | Observed Power^a^ |
| fatige | Linear | .866 | 1 | .866 | .003 | .958 | .000 | .003 | .050 |
| fatige * Groups | Linear | 83.194 | 1 | 83.194 | .265 | .609 | .006 | .265 | .080 |
| Error(fatige) | Linear | 14420.124 | 46 | 313.481 |  |  |  |  |  |
| a. Computed using alpha = .05 | | | | | | | | | |

| **Tests of Between-Subjects Effects** | | | | | | | | |
| --- | --- | --- | --- | --- | --- | --- | --- | --- |
| Measure: MEASURE_1 | | | | | | | | |
| Transformed Variable: Average | | | | | | | | |
| Source | Type III Sum of Squares | df | Mean Square | F | Sig. | Partial Eta Squared | Noncent. Parameter | Observed Power^a^ |
| Intercept | 50715.303 | 1 | 50715.303 | 148.668 | .000 | .764 | 148.668 | 1.000 |
| Groups | 92.706 | 1 | 92.706 | .272 | .605 | .006 | .272 | .080 |
| Error | 15691.985 | 46 | 341.130 |  |  |  |  |  |
| a. Computed using alpha = .05 | | | | | | | | |

| **Parameter Estimates** | | | | | | | | | | |
| --- | --- | --- | --- | --- | --- | --- | --- | --- | --- | --- |
| Dependent Variable | Parameter | B | Std. Error | t | Sig. | 95% Confidence Interval | | Partial Eta Squared | Noncent. Parameter | Observed Power^b^ |
|  |  |  |  |  |  | Lower Bound | Upper Bound |  |  |  |
| nGClrPreA | Intercept | 22.941 | 3.220 | 7.124 | .000 | 16.459 | 29.424 | .525 | 7.124 | 1.000 |
|  | [Groups=1.00] | -.104 | 4.554 | -.023 | .982 | -9.271 | 9.064 | .000 | .023 | .050 |
|  | [Groups=2.00] | 0^a^ | . | . | . | . | . | . | . | . |
| nGClrPosA | Intercept | 24.993 | 4.111 | 6.079 | .000 | 16.717 | 33.269 | .445 | 6.079 | 1.000 |
|  | [Groups=1.00] | -3.827 | 5.814 | -.658 | .514 | -15.531 | 7.877 | .009 | .658 | .099 |
|  | [Groups=2.00] | 0^a^ | . | . | . | . | . | . | . | . |
| a. This parameter is set to zero because it is redundant. | | | | | | | | | | |
| b. Computed using alpha = .05 | | | | | | | | | | |

VL-Early stance

| **Within-Subjects Factors** | |
| --- | --- |
| Measure: MEASURE_1 | |
| fatige | Dependent Variable |
| 1 | nVLlrPreA |
| 2 | nVLlrPosA |

| **Between-Subjects Factors** | | | |
| --- | --- | --- | --- |
|  | | Value Label | N |
| Groups | 1.00 | noramweieght | 24 |
|  | 2.00 | over weight | 24 |

| **Descriptive Statistics** | | | | |
| --- | --- | --- | --- | --- |
|  | Groups | Mean | Std. Deviation | N |
| nVLlrPreA | noramweieght | 59.8953 | 50.50368 | 24 |
|  | over weight | 61.8553 | 53.93664 | 24 |
|  | Total | 60.8753 | 57.00540 | 48 |
| nVLlrPosA | noramweieght | 77.1023 | 53.94060 | 24 |
|  | over weight | 76.7775 | 73.99711 | 24 |
|  | Total | 76.9399 | 64.05781 | 48 |

| **Multivariate Tests^a^** | | | | | | | | | |
| --- | --- | --- | --- | --- | --- | --- | --- | --- | --- |
| Effect | | Value | F | Hypothesis df | Error df | Sig. | Partial Eta Squared | Noncent. Parameter | Observed Power^c^ |
| fatige | Pillai's Trace | .078 | 3.887^b^ | 1.000 | 46.000 | .055 | .078 | 3.887 | .488 |
|  | Wilks' Lambda | .922 | 3.887^b^ | 1.000 | 46.000 | .055 | .078 | 3.887 | .488 |
|  | Hotelling's Trace | .084 | 3.887^b^ | 1.000 | 46.000 | .055 | .078 | 3.887 | .488 |
|  | Roy's Largest Root | .084 | 3.887^b^ | 1.000 | 46.000 | .055 | .078 | 3.887 | .488 |
| fatige * Groups | Pillai's Trace | .000 | .020^b^ | 1.000 | 46.000 | .889 | .000 | .020 | .052 |
|  | Wilks' Lambda | 1.000 | .020^b^ | 1.000 | 46.000 | .889 | .000 | .020 | .052 |
|  | Hotelling's Trace | .000 | .020^b^ | 1.000 | 46.000 | .889 | .000 | .020 | .052 |
|  | Roy's Largest Root | .000 | .020^b^ | 1.000 | 46.000 | .889 | .000 | .020 | .052 |
| a. Design: Intercept + Groups  Within Subjects Design: fatige | | | | | | | | | |
| b. Exact statistic | | | | | | | | | |
| c. Computed using alpha = .05 | | | | | | | | | |

| **Tests of Within-Subjects Effects** | | | | | | | | | |
| --- | --- | --- | --- | --- | --- | --- | --- | --- | --- |
| Measure: MEASURE_1 | | | | | | | | | |
| Source | | Type III Sum of Squares | df | Mean Square | F | Sig. | Partial Eta Squared | Noncent. Parameter | Observed Power^a^ |
| fatige | Sphericity Assumed | 6193.766 | 1 | 6193.766 | 3.887 | .055 | .078 | 3.887 | .488 |
|  | Greenhouse-Geisser | 6193.766 | 1.000 | 6193.766 | 3.887 | .055 | .078 | 3.887 | .488 |
|  | Huynh-Feldt | 6193.766 | 1.000 | 6193.766 | 3.887 | .055 | .078 | 3.887 | .488 |
|  | Lower-bound | 6193.766 | 1.000 | 6193.766 | 3.887 | .055 | .078 | 3.887 | .488 |
| fatige * Groups | Sphericity Assumed | 31.323 | 1 | 31.323 | .020 | .889 | .000 | .020 | .052 |
|  | Greenhouse-Geisser | 31.323 | 1.000 | 31.323 | .020 | .889 | .000 | .020 | .052 |
|  | Huynh-Feldt | 31.323 | 1.000 | 31.323 | .020 | .889 | .000 | .020 | .052 |
|  | Lower-bound | 31.323 | 1.000 | 31.323 | .020 | .889 | .000 | .020 | .052 |
| Error(fatige) | Sphericity Assumed | 73301.447 | 46 | 1593.510 |  |  |  |  |  |
|  | Greenhouse-Geisser | 73301.447 | 46.000 | 1593.510 |  |  |  |  |  |
|  | Huynh-Feldt | 73301.447 | 46.000 | 1593.510 |  |  |  |  |  |
|  | Lower-bound | 73301.447 | 46.000 | 1593.510 |  |  |  |  |  |
| a. Computed using alpha = .05 | | | | | | | | | |

| **Tests of Within-Subjects Contrasts** | | | | | | | | | |
| --- | --- | --- | --- | --- | --- | --- | --- | --- | --- |
| Measure: MEASURE_1 | | | | | | | | | |
| Source | fatige | Type III Sum of Squares | df | Mean Square | F | Sig. | Partial Eta Squared | Noncent. Parameter | Observed Power^a^ |
| fatige | Linear | 6193.766 | 1 | 6193.766 | 3.887 | .055 | .078 | 3.887 | .488 |
| fatige * Groups | Linear | 31.323 | 1 | 31.323 | .020 | .889 | .000 | .020 | .052 |
| Error(fatige) | Linear | 73301.447 | 46 | 1593.510 |  |  |  |  |  |
| a. Computed using alpha = .05 | | | | | | | | | |

| **Tests of Between-Subjects Effects** | | | | | | | | |
| --- | --- | --- | --- | --- | --- | --- | --- | --- |
| Measure: MEASURE_1 | | | | | | | | |
| Transformed Variable: Average | | | | | | | | |
| Source | Type III Sum of Squares | df | Mean Square | F | Sig. | Partial Eta Squared | Noncent. Parameter | Observed Power^a^ |
| Intercept | 455832.620 | 1 | 455832.620 | 77.021 | .000 | .626 | 77.021 | 1.000 |
| Groups | 16.043 | 1 | 16.043 | .003 | .959 | .000 | .003 | .050 |
| Error | 272243.100 | 46 | 5918.328 |  |  |  |  |  |
| a. Computed using alpha = .05 | | | | | | | | |

| **Parameter Estimates** | | | | | | | | | | |
| --- | --- | --- | --- | --- | --- | --- | --- | --- | --- | --- |
| Dependent Variable | Parameter | B | Std. Error | t | Sig. | 95% Confidence Interval | | Partial Eta Squared | Noncent. Parameter | Observed Power^b^ |
|  |  |  |  |  |  | Lower Bound | Upper Bound |  |  |  |
| nVLlrPreA | Intercept | 61.855 | 11.760 | 5.260 | .000 | 38.183 | 85.527 | .376 | 5.260 | .999 |
|  | [Groups=1.00] | -1.960 | 16.631 | -.118 | .907 | -35.437 | 31.517 | .000 | .118 | .052 |
|  | [Groups=2.00] | 0^a^ | . | . | . | . | . | . | . | . |
| nVLlrPosA | Intercept | 76.778 | 13.217 | 5.809 | .000 | 50.173 | 103.382 | .423 | 5.809 | 1.000 |
|  | [Groups=1.00] | .325 | 18.692 | .017 | .986 | -37.300 | 37.949 | .000 | .017 | .050 |
|  | [Groups=2.00] | 0^a^ | . | . | . | . | . | . | . | . |
| a. This parameter is set to zero because it is redundant. | | | | | | | | | | |
| b. Computed using alpha = .05 | | | | | | | | | | |

VM- Early stance phase

**General Linear Model**

| **Within-Subjects Factors** | |
| --- | --- |
| Measure: MEASURE_1 | |
| fatige | Dependent Variable |
| 1 | nVMlrPreA |
| 2 | nVMlrPosA |

| **Between-Subjects Factors** | | | |
| --- | --- | --- | --- |
|  | | Value Label | N |
| Groups | 1.00 | noramweieght | 24 |
|  | 2.00 | over weight | 24 |

| **Descriptive Statistics** | | | | |
| --- | --- | --- | --- | --- |
|  | Groups | Mean | Std. Deviation | N |
| nVMlrPreA | noramweieght | 48.2189 | 35.20702 | 24 |
|  | over weight | 37.3660 | 16.01626 | 24 |
|  | Total | 42.7924 | 27.60769 | 48 |
| nVMlrPosA | noramweieght | 52.2752 | 33.89938 | 24 |
|  | over weight | 64.3216 | 58.27866 | 24 |
|  | Total | 58.2984 | 47.55501 | 48 |

| **Multivariate Tests^a^** | | | | | | | | | |
| --- | --- | --- | --- | --- | --- | --- | --- | --- | --- |
| Effect | | Value | F | Hypothesis df | Error df | Sig. | Partial Eta Squared | Noncent. Parameter | Observed Power^c^ |
| fatige | Pillai's Trace | .078 | 3.884^b^ | 1.000 | 46.000 | .055 | .078 | 3.884 | .488 |
|  | Wilks' Lambda | .922 | 3.884^b^ | 1.000 | 46.000 | .055 | .078 | 3.884 | .488 |
|  | Hotelling's Trace | .084 | 3.884^b^ | 1.000 | 46.000 | .055 | .078 | 3.884 | .488 |
|  | Roy's Largest Root | .084 | 3.884^b^ | 1.000 | 46.000 | .055 | .078 | 3.884 | .488 |
| fatige * Groups | Pillai's Trace | .044 | 2.118^b^ | 1.000 | 46.000 | .152 | .044 | 2.118 | .297 |
|  | Wilks' Lambda | .956 | 2.118^b^ | 1.000 | 46.000 | .152 | .044 | 2.118 | .297 |
|  | Hotelling's Trace | .046 | 2.118^b^ | 1.000 | 46.000 | .152 | .044 | 2.118 | .297 |
|  | Roy's Largest Root | .046 | 2.118^b^ | 1.000 | 46.000 | .152 | .044 | 2.118 | .297 |
| a. Design: Intercept + Groups  Within Subjects Design: fatige | | | | | | | | | |
| b. Exact statistic | | | | | | | | | |
| c. Computed using alpha = .05 | | | | | | | | | |

| **Tests of Within-Subjects Effects** | | | | | | | | | |
| --- | --- | --- | --- | --- | --- | --- | --- | --- | --- |
| Measure: MEASURE_1 | | | | | | | | | |
| Source | | Type III Sum of Squares | df | Mean Square | F | Sig. | Partial Eta Squared | Noncent. Parameter | Observed Power^a^ |
| fatige | Sphericity Assumed | 5770.426 | 1 | 5770.426 | 3.884 | .055 | .078 | 3.884 | .488 |
|  | Greenhouse-Geisser | 5770.426 | 1.000 | 5770.426 | 3.884 | .055 | .078 | 3.884 | .488 |
|  | Huynh-Feldt | 5770.426 | 1.000 | 5770.426 | 3.884 | .055 | .078 | 3.884 | .488 |
|  | Lower-bound | 5770.426 | 1.000 | 5770.426 | 3.884 | .055 | .078 | 3.884 | .488 |
| fatige * Groups | Sphericity Assumed | 3146.259 | 1 | 3146.259 | 2.118 | .152 | .044 | 2.118 | .297 |
|  | Greenhouse-Geisser | 3146.259 | 1.000 | 3146.259 | 2.118 | .152 | .044 | 2.118 | .297 |
|  | Huynh-Feldt | 3146.259 | 1.000 | 3146.259 | 2.118 | .152 | .044 | 2.118 | .297 |
|  | Lower-bound | 3146.259 | 1.000 | 3146.259 | 2.118 | .152 | .044 | 2.118 | .297 |
| Error(fatige) | Sphericity Assumed | 68344.352 | 46 | 1485.747 |  |  |  |  |  |
|  | Greenhouse-Geisser | 68344.352 | 46.000 | 1485.747 |  |  |  |  |  |
|  | Huynh-Feldt | 68344.352 | 46.000 | 1485.747 |  |  |  |  |  |
|  | Lower-bound | 68344.352 | 46.000 | 1485.747 |  |  |  |  |  |
| a. Computed using alpha = .05 | | | | | | | | | |

| **Tests of Within-Subjects Contrasts** | | | | | | | | | |
| --- | --- | --- | --- | --- | --- | --- | --- | --- | --- |
| Measure: MEASURE_1 | | | | | | | | | |
| Source | fatige | Type III Sum of Squares | df | Mean Square | F | Sig. | Partial Eta Squared | Noncent. Parameter | Observed Power^a^ |
| fatige | Linear | 5770.426 | 1 | 5770.426 | 3.884 | .055 | .078 | 3.884 | .488 |
| fatige * Groups | Linear | 3146.259 | 1 | 3146.259 | 2.118 | .152 | .044 | 2.118 | .297 |
| Error(fatige) | Linear | 68344.352 | 46 | 1485.747 |  |  |  |  |  |
| a. Computed using alpha = .05 | | | | | | | | | |

| **Tests of Between-Subjects Effects** | | | | | | | | |
| --- | --- | --- | --- | --- | --- | --- | --- | --- |
| Measure: MEASURE_1 | | | | | | | | |
| Transformed Variable: Average | | | | | | | | |
| Source | Type III Sum of Squares | df | Mean Square | F | Sig. | Partial Eta Squared | Noncent. Parameter | Observed Power^a^ |
| Intercept | 245264.466 | 1 | 245264.466 | 159.775 | .000 | .776 | 159.775 | 1.000 |
| Groups | 8.547 | 1 | 8.547 | .006 | .941 | .000 | .006 | .051 |
| Error | 70613.011 | 46 | 1535.065 |  |  |  |  |  |
| a. Computed using alpha = .05 | | | | | | | | |

| **Parameter Estimates** | | | | | | | | | | |
| --- | --- | --- | --- | --- | --- | --- | --- | --- | --- | --- |
| Dependent Variable | Parameter | B | Std. Error | t | Sig. | 95% Confidence Interval | | Partial Eta Squared | Noncent. Parameter | Observed Power^b^ |
|  |  |  |  |  |  | Lower Bound | Upper Bound |  |  |  |
| nVMlrPreA | Intercept | 37.366 | 5.583 | 6.693 | .000 | 26.128 | 48.604 | .493 | 6.693 | 1.000 |
|  | [Groups=1.00] | 10.853 | 7.895 | 1.375 | .176 | -5.040 | 26.745 | .039 | 1.375 | .270 |
|  | [Groups=2.00] | 0^a^ | . | . | . | . | . | . | . | . |
| nVMlrPosA | Intercept | 64.322 | 9.731 | 6.610 | .000 | 44.733 | 83.910 | .487 | 6.610 | 1.000 |
|  | [Groups=1.00] | -12.046 | 13.762 | -.875 | .386 | -39.748 | 15.656 | .016 | .875 | .137 |
|  | [Groups=2.00] | 0^a^ | . | . | . | . | . | . | . | . |
| a. This parameter is set to zero because it is redundant. | | | | | | | | | | |
| b. Computed using alpha = .05 | | | | | | | | | | |

RF- Early stance phase

| **Within-Subjects Factors** | |
| --- | --- |
| Measure: MEASURE_1 | |
| fatige | Dependent Variable |
| 1 | nRFlrPreA |
| 2 | nRFlrPosA |

| **Between-Subjects Factors** | | | |
| --- | --- | --- | --- |
|  | | Value Label | N |
| Groups | 1.00 | noramweieght | 24 |
|  | 2.00 | over weight | 24 |

| **Descriptive Statistics** | | | | |
| --- | --- | --- | --- | --- |
|  | Groups | Mean | Std. Deviation | N |
| nRFlrPreA | noramweieght | 29.5940 | 14.02600 | 24 |
|  | over weight | 35.8083 | 20.05960 | 24 |
|  | Total | 32.7011 | 17.40818 | 48 |
| nRFlrPosA | noramweieght | 48.2233 | 74.75358 | 24 |
|  | over weight | 57.6456 | 44.46767 | 24 |
|  | Total | 52.9344 | 61.03212 | 48 |

| **Box's Test of Equality of Covariance Matrices^a^** | |
| --- | --- |
| Box's M | 9.503 |
| F | 3.018 |
| df1 | 3 |
| df2 | 380880.000 |
| Sig. | .029 |
| Tests the null hypothesis that the observed covariance matrices of the dependent variables are equal across groups. | |
| a. Design: Intercept + Groups  Within Subjects Design: fatige | |

| **Multivariate Tests^a^** | | | | | | | | | |
| --- | --- | --- | --- | --- | --- | --- | --- | --- | --- |
| Effect | | Value | F | Hypothesis df | Error df | Sig. | Partial Eta Squared | Noncent. Parameter | Observed Power^c^ |
| fatige | Pillai's Trace | .103 | 5.284^b^ | 1.000 | 46.000 | .026 | .103 | 5.284 | .614 |
|  | Wilks' Lambda | .897 | 5.284^b^ | 1.000 | 46.000 | .026 | .103 | 5.284 | .614 |
|  | Hotelling's Trace | .115 | 5.284^b^ | 1.000 | 46.000 | .026 | .103 | 5.284 | .614 |
|  | Roy's Largest Root | .115 | 5.284^b^ | 1.000 | 46.000 | .026 | .103 | 5.284 | .614 |
| fatige * Groups | Pillai's Trace | .001 | .033^b^ | 1.000 | 46.000 | .856 | .001 | .033 | .054 |
|  | Wilks' Lambda | .999 | .033^b^ | 1.000 | 46.000 | .856 | .001 | .033 | .054 |
|  | Hotelling's Trace | .001 | .033^b^ | 1.000 | 46.000 | .856 | .001 | .033 | .054 |
|  | Roy's Largest Root | .001 | .033^b^ | 1.000 | 46.000 | .856 | .001 | .033 | .054 |
| a. Design: Intercept + Groups  Within Subjects Design: fatige | | | | | | | | | |
| b. Exact statistic | | | | | | | | | |
| c. Computed using alpha = .05 | | | | | | | | | |

| **Tests of Within-Subjects Effects** | | | | | | | | | |
| --- | --- | --- | --- | --- | --- | --- | --- | --- | --- |
| Measure: MEASURE_1 | | | | | | | | | |
| Source | | Type III Sum of Squares | df | Mean Square | F | Sig. | Partial Eta Squared | Noncent. Parameter | Observed Power^a^ |
| fatige | Sphericity Assumed | 9825.264 | 1 | 9825.264 | 5.284 | .026 | .103 | 5.284 | .614 |
|  | Greenhouse-Geisser | 9825.264 | 1.000 | 9825.264 | 5.284 | .026 | .103 | 5.284 | .614 |
|  | Huynh-Feldt | 9825.264 | 1.000 | 9825.264 | 5.284 | .026 | .103 | 5.284 | .614 |
|  | Lower-bound | 9825.264 | 1.000 | 9825.264 | 5.284 | .026 | .103 | 5.284 | .614 |
| fatige * Groups | Sphericity Assumed | 61.745 | 1 | 61.745 | .033 | .856 | .001 | .033 | .054 |
|  | Greenhouse-Geisser | 61.745 | 1.000 | 61.745 | .033 | .856 | .001 | .033 | .054 |
|  | Huynh-Feldt | 61.745 | 1.000 | 61.745 | .033 | .856 | .001 | .033 | .054 |
|  | Lower-bound | 61.745 | 1.000 | 61.745 | .033 | .856 | .001 | .033 | .054 |
| Error(fatige) | Sphericity Assumed | 85528.670 | 46 | 1859.319 |  |  |  |  |  |
|  | Greenhouse-Geisser | 85528.670 | 46.000 | 1859.319 |  |  |  |  |  |
|  | Huynh-Feldt | 85528.670 | 46.000 | 1859.319 |  |  |  |  |  |
|  | Lower-bound | 85528.670 | 46.000 | 1859.319 |  |  |  |  |  |
| a. Computed using alpha = .05 | | | | | | | | | |

| **Tests of Within-Subjects Contrasts** | | | | | | | | | |
| --- | --- | --- | --- | --- | --- | --- | --- | --- | --- |
| Measure: MEASURE_1 | | | | | | | | | |
| Source | fatige | Type III Sum of Squares | df | Mean Square | F | Sig. | Partial Eta Squared | Noncent. Parameter | Observed Power^a^ |
| fatige | Linear | 9825.264 | 1 | 9825.264 | 5.284 | .026 | .103 | 5.284 | .614 |
| fatige * Groups | Linear | 61.745 | 1 | 61.745 | .033 | .856 | .001 | .033 | .054 |
| Error(fatige) | Linear | 85528.670 | 46 | 1859.319 |  |  |  |  |  |
| a. Computed using alpha = .05 | | | | | | | | | |

| **Tests of Between-Subjects Effects** | | | | | | | | |
| --- | --- | --- | --- | --- | --- | --- | --- | --- |
| Measure: MEASURE_1 | | | | | | | | |
| Transformed Variable: Average | | | | | | | | |
| Source | Type III Sum of Squares | df | Mean Square | F | Sig. | Partial Eta Squared | Noncent. Parameter | Observed Power^a^ |
| Intercept | 176002.843 | 1 | 176002.843 | 79.174 | .000 | .633 | 79.174 | 1.000 |
| Groups | 1467.022 | 1 | 1467.022 | .660 | .421 | .014 | .660 | .125 |
| Error | 102256.867 | 46 | 2222.975 |  |  |  |  |  |
| a. Computed using alpha = .05 | | | | | | | | |

| **Parameter Estimates** | | | | | | | | | | |
| --- | --- | --- | --- | --- | --- | --- | --- | --- | --- | --- |
| Dependent Variable | Parameter | B | Std. Error | t | Sig. | 95% Confidence Interval | | Partial Eta Squared | Noncent. Parameter | Observed Power^b^ |
|  |  |  |  |  |  | Lower Bound | Upper Bound |  |  |  |
| nRFlrPreA | Intercept | 35.808 | 3.533 | 10.136 | .000 | 28.697 | 42.920 | .691 | 10.136 | 1.000 |
|  | [Groups=1.00] | -6.214 | 4.996 | -1.244 | .220 | -16.271 | 3.843 | .033 | 1.244 | .230 |
|  | [Groups=2.00] | 0^a^ | . | . | . | . | . | . | . | . |
| nRFlrPosA | Intercept | 57.646 | 12.554 | 4.592 | .000 | 32.375 | 82.916 | .314 | 4.592 | .994 |
|  | [Groups=1.00] | -9.422 | 17.755 | -.531 | .598 | -45.161 | 26.316 | .006 | .531 | .081 |
|  | [Groups=2.00] | 0^a^ | . | . | . | . | . | . | . | . |
| a. This parameter is set to zero because it is redundant. | | | | | | | | | | |
| b. Computed using alpha = .05 | | | | | | | | | | |

BF- Early stance phase

| **Within-Subjects Factors** | |
| --- | --- |
| Measure: MEASURE_1 | |
| fatige | Dependent Variable |
| 1 | nBFlrPreA |
| 2 | nBFlrPosA |

| **Between-Subjects Factors** | | | |
| --- | --- | --- | --- |
|  | | Value Label | N |
| Groups | 1.00 | noramweieght | 24 |
|  | 2.00 | over weight | 24 |

| **Descriptive Statistics** | | | | |
| --- | --- | --- | --- | --- |
|  | Groups | Mean | Std. Deviation | N |
| nBFlrPreA | noramweieght | 29.2318 | 10.21608 | 24 |
|  | over weight | 44.3587 | 28.84389 | 24 |
|  | Total | 36.7953 | 22.72952 | 48 |
| nBFlrPosA | noramweieght | 41.4210 | 41.12024 | 24 |
|  | over weight | 48.5184 | 24.31399 | 24 |
|  | Total | 44.9697 | 33.60961 | 48 |

| **Multivariate Tests^a^** | | | | | | | | | |
| --- | --- | --- | --- | --- | --- | --- | --- | --- | --- |
| Effect | | Value | F | Hypothesis df | Error df | Sig. | Partial Eta Squared | Noncent. Parameter | Observed Power^c^ |
| fatige | Pillai's Trace | .067 | 3.292^b^ | 1.000 | 46.000 | .076 | .067 | 3.292 | .427 |
|  | Wilks' Lambda | .933 | 3.292^b^ | 1.000 | 46.000 | .076 | .067 | 3.292 | .427 |
|  | Hotelling's Trace | .072 | 3.292^b^ | 1.000 | 46.000 | .076 | .067 | 3.292 | .427 |
|  | Roy's Largest Root | .072 | 3.292^b^ | 1.000 | 46.000 | .076 | .067 | 3.292 | .427 |
| fatige * Groups | Pillai's Trace | .017 | .794^b^ | 1.000 | 46.000 | .378 | .017 | .794 | .141 |
|  | Wilks' Lambda | .983 | .794^b^ | 1.000 | 46.000 | .378 | .017 | .794 | .141 |
|  | Hotelling's Trace | .017 | .794^b^ | 1.000 | 46.000 | .378 | .017 | .794 | .141 |
|  | Roy's Largest Root | .017 | .794^b^ | 1.000 | 46.000 | .378 | .017 | .794 | .141 |
| a. Design: Intercept + Groups  Within Subjects Design: fatige | | | | | | | | | |
| b. Exact statistic | | | | | | | | | |
| c. Computed using alpha = .05 | | | | | | | | | |

| **Tests of Within-Subjects Effects** | | | | | | | | | |
| --- | --- | --- | --- | --- | --- | --- | --- | --- | --- |
| Measure: MEASURE_1 | | | | | | | | | |
| Source | | Type III Sum of Squares | df | Mean Square | F | Sig. | Partial Eta Squared | Noncent. Parameter | Observed Power^a^ |
| fatige | Sphericity Assumed | 1603.713 | 1 | 1603.713 | 3.292 | .076 | .067 | 3.292 | .427 |
|  | Greenhouse-Geisser | 1603.713 | 1.000 | 1603.713 | 3.292 | .076 | .067 | 3.292 | .427 |
|  | Huynh-Feldt | 1603.713 | 1.000 | 1603.713 | 3.292 | .076 | .067 | 3.292 | .427 |
|  | Lower-bound | 1603.713 | 1.000 | 1603.713 | 3.292 | .076 | .067 | 3.292 | .427 |
| fatige * Groups | Sphericity Assumed | 386.842 | 1 | 386.842 | .794 | .378 | .017 | .794 | .141 |
|  | Greenhouse-Geisser | 386.842 | 1.000 | 386.842 | .794 | .378 | .017 | .794 | .141 |
|  | Huynh-Feldt | 386.842 | 1.000 | 386.842 | .794 | .378 | .017 | .794 | .141 |
|  | Lower-bound | 386.842 | 1.000 | 386.842 | .794 | .378 | .017 | .794 | .141 |
| Error(fatige) | Sphericity Assumed | 22410.699 | 46 | 487.189 |  |  |  |  |  |
|  | Greenhouse-Geisser | 22410.699 | 46.000 | 487.189 |  |  |  |  |  |
|  | Huynh-Feldt | 22410.699 | 46.000 | 487.189 |  |  |  |  |  |
|  | Lower-bound | 22410.699 | 46.000 | 487.189 |  |  |  |  |  |
| a. Computed using alpha = .05 | | | | | | | | | |

| **Tests of Within-Subjects Contrasts** | | | | | | | | | |
| --- | --- | --- | --- | --- | --- | --- | --- | --- | --- |
| Measure: MEASURE_1 | | | | | | | | | |
| Source | fatige | Type III Sum of Squares | df | Mean Square | F | Sig. | Partial Eta Squared | Noncent. Parameter | Observed Power^a^ |
| fatige | Linear | 1603.713 | 1 | 1603.713 | 3.292 | .076 | .067 | 3.292 | .427 |
| fatige * Groups | Linear | 386.842 | 1 | 386.842 | .794 | .378 | .017 | .794 | .141 |
| Error(fatige) | Linear | 22410.699 | 46 | 487.189 |  |  |  |  |  |
| a. Computed using alpha = .05 | | | | | | | | | |

| **Tests of Between-Subjects Effects** | | | | | | | | |
| --- | --- | --- | --- | --- | --- | --- | --- | --- |
| Measure: MEASURE_1 | | | | | | | | |
| Transformed Variable: Average | | | | | | | | |
| Source | Type III Sum of Squares | df | Mean Square | F | Sig. | Partial Eta Squared | Noncent. Parameter | Observed Power^a^ |
| Intercept | 160452.327 | 1 | 160452.327 | 143.005 | .000 | .757 | 143.005 | 1.000 |
| Groups | 2963.504 | 1 | 2963.504 | 2.641 | .111 | .054 | 2.641 | .356 |
| Error | 51612.096 | 46 | 1122.002 |  |  |  |  |  |
| a. Computed using alpha = .05 | | | | | | | | |

| **Parameter Estimates** | | | | | | | | | | |
| --- | --- | --- | --- | --- | --- | --- | --- | --- | --- | --- |
| Dependent Variable | Parameter | B | Std. Error | t | Sig. | 95% Confidence Interval | | Partial Eta Squared | Noncent. Parameter | Observed Power^b^ |
|  |  |  |  |  |  | Lower Bound | Upper Bound |  |  |  |
| nBFlrPreA | Intercept | 44.359 | 4.417 | 10.043 | .000 | 35.468 | 53.249 | .687 | 10.043 | 1.000 |
|  | [Groups=1.00] | -15.127 | 6.246 | -2.422 | .019 | -27.700 | -2.554 | .113 | 2.422 | .659 |
|  | [Groups=2.00] | 0^a^ | . | . | . | . | . | . | . | . |
| nBFlrPosA | Intercept | 48.518 | 6.895 | 7.037 | .000 | 34.639 | 62.398 | .518 | 7.037 | 1.000 |
|  | [Groups=1.00] | -7.097 | 9.751 | -.728 | .470 | -26.725 | 12.531 | .011 | .728 | .110 |
|  | [Groups=2.00] | 0^a^ | . | . | . | . | . | . | . | . |
| a. This parameter is set to zero because it is redundant. | | | | | | | | | | |
| b. Computed using alpha = .05 | | | | | | | | | | |

ST- Early stance phase

| **Within-Subjects Factors** | |
| --- | --- |
| Measure: MEASURE_1 | |
| fatige | Dependent Variable |
| 1 | nSTlrPreA |
| 2 | nSTlrPosA |

| **Between-Subjects Factors** | | | |
| --- | --- | --- | --- |
|  | | Value Label | N |
| Groups | 1.00 | noramweieght | 24 |
|  | 2.00 | over weight | 24 |

| **Descriptive Statistics** | | | | |
| --- | --- | --- | --- | --- |
|  | Groups | Mean | Std. Deviation | N |
| nSTlrPreA | noramweieght | 38.8890 | 27.96172 | 24 |
|  | over weight | 44.9046 | 27.53087 | 24 |
|  | Total | 41.8968 | 27.61814 | 48 |
| nSTlrPosA | noramweieght | 57.1337 | 46.59818 | 24 |
|  | over weight | 73.7579 | 57.48905 | 24 |
|  | Total | 65.4458 | 52.44513 | 48 |

| **Multivariate Tests^a^** | | | | | | | | | |
| --- | --- | --- | --- | --- | --- | --- | --- | --- | --- |
| Effect | | Value | F | Hypothesis df | Error df | Sig. | Partial Eta Squared | Noncent. Parameter | Observed Power^c^ |
| fatige | Pillai's Trace | .137 | 7.292^b^ | 1.000 | 46.000 | .010 | .137 | 7.292 | .753 |
|  | Wilks' Lambda | .863 | 7.292^b^ | 1.000 | 46.000 | .010 | .137 | 7.292 | .753 |
|  | Hotelling's Trace | .159 | 7.292^b^ | 1.000 | 46.000 | .010 | .137 | 7.292 | .753 |
|  | Roy's Largest Root | .159 | 7.292^b^ | 1.000 | 46.000 | .010 | .137 | 7.292 | .753 |
| fatige * Groups | Pillai's Trace | .008 | .370^b^ | 1.000 | 46.000 | .546 | .008 | .370 | .092 |
|  | Wilks' Lambda | .992 | .370^b^ | 1.000 | 46.000 | .546 | .008 | .370 | .092 |
|  | Hotelling's Trace | .008 | .370^b^ | 1.000 | 46.000 | .546 | .008 | .370 | .092 |
|  | Roy's Largest Root | .008 | .370^b^ | 1.000 | 46.000 | .546 | .008 | .370 | .092 |
| a. Design: Intercept + Groups  Within Subjects Design: fatige | | | | | | | | | |
| b. Exact statistic | | | | | | | | | |
| c. Computed using alpha = .05 | | | | | | | | | |

| **Tests of Within-Subjects Effects** | | | | | | | | | |
| --- | --- | --- | --- | --- | --- | --- | --- | --- | --- |
| Measure: MEASURE_1 | | | | | | | | | |
| Source | | Type III Sum of Squares | df | Mean Square | F | Sig. | Partial Eta Squared | Noncent. Parameter | Observed Power^a^ |
| fatige | Sphericity Assumed | 13309.318 | 1 | 13309.318 | 7.292 | .010 | .137 | 7.292 | .753 |
|  | Greenhouse-Geisser | 13309.318 | 1.000 | 13309.318 | 7.292 | .010 | .137 | 7.292 | .753 |
|  | Huynh-Feldt | 13309.318 | 1.000 | 13309.318 | 7.292 | .010 | .137 | 7.292 | .753 |
|  | Lower-bound | 13309.318 | 1.000 | 13309.318 | 7.292 | .010 | .137 | 7.292 | .753 |
| fatige * Groups | Sphericity Assumed | 675.255 | 1 | 675.255 | .370 | .546 | .008 | .370 | .092 |
|  | Greenhouse-Geisser | 675.255 | 1.000 | 675.255 | .370 | .546 | .008 | .370 | .092 |
|  | Huynh-Feldt | 675.255 | 1.000 | 675.255 | .370 | .546 | .008 | .370 | .092 |
|  | Lower-bound | 675.255 | 1.000 | 675.255 | .370 | .546 | .008 | .370 | .092 |
| Error(fatige) | Sphericity Assumed | 83964.415 | 46 | 1825.313 |  |  |  |  |  |
|  | Greenhouse-Geisser | 83964.415 | 46.000 | 1825.313 |  |  |  |  |  |
|  | Huynh-Feldt | 83964.415 | 46.000 | 1825.313 |  |  |  |  |  |
|  | Lower-bound | 83964.415 | 46.000 | 1825.313 |  |  |  |  |  |
| a. Computed using alpha = .05 | | | | | | | | | |

| **Tests of Within-Subjects Contrasts** | | | | | | | | | |
| --- | --- | --- | --- | --- | --- | --- | --- | --- | --- |
| Measure: MEASURE_1 | | | | | | | | | |
| Source | fatige | Type III Sum of Squares | df | Mean Square | F | Sig. | Partial Eta Squared | Noncent. Parameter | Observed Power^a^ |
| fatige | Linear | 13309.318 | 1 | 13309.318 | 7.292 | .010 | .137 | 7.292 | .753 |
| fatige * Groups | Linear | 675.255 | 1 | 675.255 | .370 | .546 | .008 | .370 | .092 |
| Error(fatige) | Linear | 83964.415 | 46 | 1825.313 |  |  |  |  |  |
| a. Computed using alpha = .05 | | | | | | | | | |

| **Tests of Between-Subjects Effects** | | | | | | | | |
| --- | --- | --- | --- | --- | --- | --- | --- | --- |
| Measure: MEASURE_1 | | | | | | | | |
| Transformed Variable: Average | | | | | | | | |
| Source | Type III Sum of Squares | df | Mean Square | F | Sig. | Partial Eta Squared | Noncent. Parameter | Observed Power^a^ |
| Intercept | 276538.394 | 1 | 276538.394 | 164.334 | .000 | .781 | 164.334 | 1.000 |
| Groups | 3075.326 | 1 | 3075.326 | 1.828 | .183 | .038 | 1.828 | .263 |
| Error | 77407.904 | 46 | 1682.781 |  |  |  |  |  |
| a. Computed using alpha = .05 | | | | | | | | |

| **Parameter Estimates** | | | | | | | | | | |
| --- | --- | --- | --- | --- | --- | --- | --- | --- | --- | --- |
| Dependent Variable | Parameter | B | Std. Error | t | Sig. | 95% Confidence Interval | | Partial Eta Squared | Noncent. Parameter | Observed Power^b^ |
|  |  |  |  |  |  | Lower Bound | Upper Bound |  |  |  |
| nSTlrPreA | Intercept | 44.905 | 5.664 | 7.928 | .000 | 33.504 | 56.305 | .577 | 7.928 | 1.000 |
|  | [Groups=1.00] | -6.016 | 8.010 | -.751 | .456 | -22.139 | 10.108 | .012 | .751 | .114 |
|  | [Groups=2.00] | 0^a^ | . | . | . | . | . | . | . | . |
| nSTlrPosA | Intercept | 73.758 | 10.681 | 6.905 | .000 | 52.257 | 95.258 | .509 | 6.905 | 1.000 |
|  | [Groups=1.00] | -16.624 | 15.106 | -1.101 | .277 | -47.030 | 13.782 | .026 | 1.101 | .190 |
|  | [Groups=2.00] | 0^a^ | . | . | . | . | . | . | . | . |
| a. This parameter is set to zero because it is redundant. | | | | | | | | | | |
| b. Computed using alpha = .05 | | | | | | | | | | |

TA- Late stance

| **Within-Subjects Factors** | |
| --- | --- |
| Measure: MEASURE_1 | |
| fatige | Dependent Variable |
| 1 | nTApoPreB |
| 2 | nTApoPOSB |

| **Between-Subjects Factors** | | | |
| --- | --- | --- | --- |
|  | | Value Label | N |
| Groups | 1.00 | noramweieght | 24 |
|  | 2.00 | over weight | 24 |

| **Descriptive Statistics** | | | | |
| --- | --- | --- | --- | --- |
|  | Groups | Mean | Std. Deviation | N |
| nTApoPreB | noramweieght | 46.3541 | 17.17095 | 24 |
|  | over weight | 49.9667 | 21.85520 | 24 |
|  | Total | 48.1988 | 19.57468 | 48 |
| nTApoPOSB | noramweieght | 50.3162 | 20.76143 | 24 |
|  | over weight | 67.8172 | 41.50032 | 24 |
|  | Total | 59.2529 | 33.84504 | 48 |

| **Multivariate Tests^a^** | | | | | | | | | |
| --- | --- | --- | --- | --- | --- | --- | --- | --- | --- |
| Effect | | Value | F | Hypothesis df | Error df | Sig. | Partial Eta Squared | Noncent. Parameter | Observed Power^c^ |
| fatige | Pillai's Trace | .087 | 4.264^b^ | 1.000 | 45.000 | .045 | .087 | 4.264 | .524 |
|  | Wilks' Lambda | .913 | 4.264^b^ | 1.000 | 45.000 | .045 | .087 | 4.264 | .524 |
|  | Hotelling's Trace | .095 | 4.264^b^ | 1.000 | 45.000 | .045 | .087 | 4.264 | .524 |
|  | Roy's Largest Root | .095 | 4.264^b^ | 1.000 | 45.000 | .045 | .087 | 4.264 | .524 |
| fatige * Groups | Pillai's Trace | .037 | 1.729^b^ | 1.000 | 45.000 | .195 | .037 | 1.729 | .251 |
|  | Wilks' Lambda | .963 | 1.729^b^ | 1.000 | 45.000 | .195 | .037 | 1.729 | .251 |
|  | Hotelling's Trace | .038 | 1.729^b^ | 1.000 | 45.000 | .195 | .037 | 1.729 | .251 |
|  | Roy's Largest Root | .038 | 1.729^b^ | 1.000 | 45.000 | .195 | .037 | 1.729 | .251 |
| a. Design: Intercept + Groups  Within Subjects Design: fatige | | | | | | | | | |
| b. Exact statistic | | | | | | | | | |
| c. Computed using alpha = .05 | | | | | | | | | |

| **Tests of Within-Subjects Effects** | | | | | | | | | |
| --- | --- | --- | --- | --- | --- | --- | --- | --- | --- |
| Measure: MEASURE_1 | | | | | | | | | |
| Source | | Type III Sum of Squares | df | Mean Square | F | Sig. | Partial Eta Squared | Noncent. Parameter | Observed Power^a^ |
| fatige | Sphericity Assumed | 2793.982 | 1 | 2793.982 | 4.264 | 0.081 | .0.066 | 4.264 | .524 |
|  | Greenhouse-Geisser | 2793.982 | 1.000 | 2793.982 | 4.264 | 0.081 | .0.066 | 4.264 | .524 |
|  | Huynh-Feldt | 2793.982 | 1.000 | 2793.982 | 4.264 | 0.081 | .0.066 | 4.264 | .524 |
|  | Lower-bound | 2793.982 | 1.000 | 2793.982 | 4.264 | 0.081 | .0.066 | 4.264 | .524 |
| fatige * Groups | Sphericity Assumed | 1132.712 | 1 | 1132.712 | 1.729 | .195 | .037 | 1.729 | .251 |
|  | Greenhouse-Geisser | 1132.712 | 1.000 | 1132.712 | 1.729 | .195 | .037 | 1.729 | .251 |
|  | Huynh-Feldt | 1132.712 | 1.000 | 1132.712 | 1.729 | .195 | .037 | 1.729 | .251 |
|  | Lower-bound | 1132.712 | 1.000 | 1132.712 | 1.729 | .195 | .037 | 1.729 | .251 |
| Error(fatige) | Sphericity Assumed | 29485.049 | 45 | 655.223 |  |  |  |  |  |
|  | Greenhouse-Geisser | 29485.049 | 45.000 | 655.223 |  |  |  |  |  |
|  | Huynh-Feldt | 29485.049 | 45.000 | 655.223 |  |  |  |  |  |
|  | Lower-bound | 29485.049 | 45.000 | 655.223 |  |  |  |  |  |
| a. Computed using alpha = .05 | | | | | | | | | |

| **Tests of Within-Subjects Contrasts** | | | | | | | | | |
| --- | --- | --- | --- | --- | --- | --- | --- | --- | --- |
| Measure: MEASURE_1 | | | | | | | | | |
| Source | fatige | Type III Sum of Squares | df | Mean Square | F | Sig. | Partial Eta Squared | Noncent. Parameter | Observed Power^a^ |
| fatige | Linear | 2793.982 | 1 | 2793.982 | 4.264 | .045 | .087 | 4.264 | .524 |
| fatige * Groups | Linear | 1132.712 | 1 | 1132.712 | 1.729 | .195 | .037 | 1.729 | .251 |
| Error(fatige) | Linear | 29485.049 | 45 | 655.223 |  |  |  |  |  |
| a. Computed using alpha = .05 | | | | | | | | | |

| **Tests of Between-Subjects Effects** | | | | | | | | |
| --- | --- | --- | --- | --- | --- | --- | --- | --- |
| Measure: MEASURE_1 | | | | | | | | |
| Transformed Variable: Average | | | | | | | | |
| Source | Type III Sum of Squares | df | Mean Square | F | Sig. | Partial Eta Squared | Noncent. Parameter | Observed Power^a^ |
| Intercept | 270072.481 | 1 | 270072.481 | 327.735 | .000 | .879 | 327.735 | 1.000 |
| Groups | 2617.778 | 1 | 2617.778 | 3.177 | .045 | .087 | 3.177 | .415 |
| Error | 37082.578 | 45 | 824.057 |  |  |  |  |  |
| a. Computed using alpha = .05 | | | | | | | | |

| **Parameter Estimates** | | | | | | | | | | |
| --- | --- | --- | --- | --- | --- | --- | --- | --- | --- | --- |
| Dependent Variable | Parameter | B | Std. Error | t | Sig. | 95% Confidence Interval | | Partial Eta Squared | Noncent. Parameter | Observed Power^b^ |
|  |  |  |  |  |  | Lower Bound | Upper Bound |  |  |  |
| nTApoPreB | Intercept | 49.967 | 4.022 | 12.423 | .000 | 41.866 | 58.068 | .774 | 12.423 | 1.000 |
|  | [Groups=1.00] | -3.613 | 5.750 | -.628 | .533 | -15.193 | 7.968 | .009 | .628 | .094 |
|  | [Groups=2.00] | 0^a^ | . | . | . | . | . | . | . | . |
| nTApoPOSB | Intercept | 67.817 | 6.742 | 10.058 | .000 | 54.238 | 81.397 | .692 | 10.058 | 1.000 |
|  | [Groups=1.00] | -17.501 | 9.638 | -1.816 | .076 | -36.913 | 1.911 | .068 | 1.816 | .428 |
|  | [Groups=2.00] | 0^a^ | . | . | . | . | . | . | . | . |
| a. This parameter is set to zero because it is redundant. | | | | | | | | | | |
| b. Computed using alpha = .05 | | | | | | | | | | |

Gas-M- Late stance

| **Within-Subjects Factors** | |
| --- | --- |
| Measure: MEASURE_1 | |
| fatige | Dependent Variable |
| 1 | nGCpoPreB |
| 2 | nGCpoPOSB |

| **Between-Subjects Factors** | | | |
| --- | --- | --- | --- |
|  | | Value Label | N |
| Groups | 1.00 | noramweieght | 24 |
|  | 2.00 | over weight | 24 |

| **Descriptive Statistics** | | | | |
| --- | --- | --- | --- | --- |
|  | Groups | Mean | Std. Deviation | N |
| nGCpoPreB | noramweieght | 145.9307 | 109.60075 | 24 |
|  | over weight | 142.4581 | 68.27288 | 24 |
|  | Total | 144.1944 | 90.34631 | 48 |
| nGCpoPOSB | noramweieght | 132.3886 | 70.88920 | 24 |
|  | over weight | 152.5074 | 62.32875 | 24 |
|  | Total | 142.4480 | 66.81042 | 48 |

| **Multivariate Tests^a^** | | | | | | | | | |
| --- | --- | --- | --- | --- | --- | --- | --- | --- | --- |
| Effect | | Value | F | Hypothesis df | Error df | Sig. | Partial Eta Squared | Noncent. Parameter | Observed Power^c^ |
| fatige | Pillai's Trace | .000 | .012^b^ | 1.000 | 46.000 | .912 | .000 | .012 | .051 |
|  | Wilks' Lambda | 1.000 | .012^b^ | 1.000 | 46.000 | .912 | .000 | .012 | .051 |
|  | Hotelling's Trace | .000 | .012^b^ | 1.000 | 46.000 | .912 | .000 | .012 | .051 |
|  | Roy's Largest Root | .000 | .012^b^ | 1.000 | 46.000 | .912 | .000 | .012 | .051 |
| fatige * Groups | Pillai's Trace | .012 | .559^b^ | 1.000 | 46.000 | .459 | .012 | .559 | .113 |
|  | Wilks' Lambda | .988 | .559^b^ | 1.000 | 46.000 | .459 | .012 | .559 | .113 |
|  | Hotelling's Trace | .012 | .559^b^ | 1.000 | 46.000 | .459 | .012 | .559 | .113 |
|  | Roy's Largest Root | .012 | .559^b^ | 1.000 | 46.000 | .459 | .012 | .559 | .113 |
| a. Design: Intercept + Groups  Within Subjects Design: fatige | | | | | | | | | |
| b. Exact statistic | | | | | | | | | |
| c. Computed using alpha = .05 | | | | | | | | | |

| **Tests of Within-Subjects Effects** | | | | | | | | | |
| --- | --- | --- | --- | --- | --- | --- | --- | --- | --- |
| Measure: MEASURE_1 | | | | | | | | | |
| Source | | Type III Sum of Squares | df | Mean Square | F | Sig. | Partial Eta Squared | Noncent. Parameter | Observed Power^a^ |
| fatige | Sphericity Assumed | 73.194 | 1 | 73.194 | .012 | .912 | .000 | .012 | .051 |
|  | Greenhouse-Geisser | 73.194 | 1.000 | 73.194 | .012 | .912 | .000 | .012 | .051 |
|  | Huynh-Feldt | 73.194 | 1.000 | 73.194 | .012 | .912 | .000 | .012 | .051 |
|  | Lower-bound | 73.194 | 1.000 | 73.194 | .012 | .912 | .000 | .012 | .051 |
| fatige * Groups | Sphericity Assumed | 3339.341 | 1 | 3339.341 | .559 | .459 | .012 | .559 | .113 |
|  | Greenhouse-Geisser | 3339.341 | 1.000 | 3339.341 | .559 | .459 | .012 | .559 | .113 |
|  | Huynh-Feldt | 3339.341 | 1.000 | 3339.341 | .559 | .459 | .012 | .559 | .113 |
|  | Lower-bound | 3339.341 | 1.000 | 3339.341 | .559 | .459 | .012 | .559 | .113 |
| Error(fatige) | Sphericity Assumed | 274990.850 | 46 | 5978.062 |  |  |  |  |  |
|  | Greenhouse-Geisser | 274990.850 | 46.000 | 5978.062 |  |  |  |  |  |
|  | Huynh-Feldt | 274990.850 | 46.000 | 5978.062 |  |  |  |  |  |
|  | Lower-bound | 274990.850 | 46.000 | 5978.062 |  |  |  |  |  |
| a. Computed using alpha = .05 | | | | | | | | | |

| **Tests of Within-Subjects Contrasts** | | | | | | | | | |
| --- | --- | --- | --- | --- | --- | --- | --- | --- | --- |
| Measure: MEASURE_1 | | | | | | | | | |
| Source | fatige | Type III Sum of Squares | df | Mean Square | F | Sig. | Partial Eta Squared | Noncent. Parameter | Observed Power^a^ |
| fatige | Linear | 73.194 | 1 | 73.194 | .012 | .912 | .000 | .012 | .051 |
| fatige * Groups | Linear | 3339.341 | 1 | 3339.341 | .559 | .459 | .012 | .559 | .113 |
| Error(fatige) | Linear | 274990.850 | 46 | 5978.062 |  |  |  |  |  |
| a. Computed using alpha = .05 | | | | | | | | | |

| **Tests of Between-Subjects Effects** | | | | | | | | |
| --- | --- | --- | --- | --- | --- | --- | --- | --- |
| Measure: MEASURE_1 | | | | | | | | |
| Transformed Variable: Average | | | | | | | | |
| Source | Type III Sum of Squares | df | Mean Square | F | Sig. | Partial Eta Squared | Noncent. Parameter | Observed Power^a^ |
| Intercept | 1971933.066 | 1 | 1971933.066 | 289.404 | .000 | .863 | 289.404 | 1.000 |
| Groups | 1662.575 | 1 | 1662.575 | .244 | .624 | .005 | .244 | .077 |
| Error | 313433.367 | 46 | 6813.769 |  |  |  |  |  |
| a. Computed using alpha = .05 | | | | | | | | |

| **Parameter Estimates** | | | | | | | | | | |
| --- | --- | --- | --- | --- | --- | --- | --- | --- | --- | --- |
| Dependent Variable | Parameter | B | Std. Error | t | Sig. | 95% Confidence Interval | | Partial Eta Squared | Noncent. Parameter | Observed Power^b^ |
|  |  |  |  |  |  | Lower Bound | Upper Bound |  |  |  |
| nGCpoPreB | Intercept | 142.458 | 18.638 | 7.644 | .000 | 104.942 | 179.974 | .559 | 7.644 | 1.000 |
|  | [Groups=1.00] | 3.473 | 26.358 | .132 | .896 | -49.583 | 56.528 | .000 | .132 | .052 |
|  | [Groups=2.00] | 0^a^ | . | . | . | . | . | . | . | . |
| nGCpoPOSB | Intercept | 152.507 | 13.625 | 11.194 | .000 | 125.083 | 179.932 | .731 | 11.194 | 1.000 |
|  | [Groups=1.00] | -20.119 | 19.268 | -1.044 | .302 | -58.903 | 18.666 | .023 | 1.044 | .176 |
|  | [Groups=2.00] | 0^a^ | . | . | . | . | . | . | . | . |
| a. This parameter is set to zero because it is redundant. | | | | | | | | | | |
| b. Computed using alpha = .05 | | | | | | | | | | |

**VL – Late stance**

| **Within-Subjects Factors** | |
| --- | --- |
| Measure: MEASURE_1 | |
| fatige | Dependent Variable |
| 1 | nVLpoPreB |
| 2 | nVLpoPOSB |

| **Between-Subjects Factors** | | | |
| --- | --- | --- | --- |
|  | | Value Label | N |
| Groups | 1.00 | noramweieght | 24 |
|  | 2.00 | over weight | 24 |

| **Descriptive Statistics** | | | | |
| --- | --- | --- | --- | --- |
|  | Groups | Mean | Std. Deviation | N |
| nVLpoPreB | noramweieght | 94.0353 | 75.30198 | 24 |
|  | over weight | 68.2228 | 27.04900 | 24 |
|  | Total | 81.1291 | 57.47196 | 48 |
| nVLpoPOSB | noramweieght | 95.5763 | 56.25308 | 24 |
|  | over weight | 122.7571 | 119.58405 | 24 |
|  | Total | 109.1667 | 93.46236 | 48 |

| **Multivariate Tests^a^** | | | | | | | | | |
| --- | --- | --- | --- | --- | --- | --- | --- | --- | --- |
| Effect | | Value | F | Hypothesis df | Error df | Sig. | Partial Eta Squared | Noncent. Parameter | Observed Power^c^ |
| fatige | Pillai's Trace | .068 | 3.351^b^ | 1.000 | 46.000 | .074 | .068 | 3.351 | .434 |
|  | Wilks' Lambda | .932 | 3.351^b^ | 1.000 | 46.000 | .074 | .068 | 3.351 | .434 |
|  | Hotelling's Trace | .073 | 3.351^b^ | 1.000 | 46.000 | .074 | .068 | 3.351 | .434 |
|  | Roy's Largest Root | .073 | 3.351^b^ | 1.000 | 46.000 | .074 | .068 | 3.351 | .434 |
| fatige * Groups | Pillai's Trace | .061 | 2.993^b^ | 1.000 | 46.000 | .090 | .061 | 2.993 | .395 |
|  | Wilks' Lambda | .939 | 2.993^b^ | 1.000 | 46.000 | .090 | .061 | 2.993 | .395 |
|  | Hotelling's Trace | .065 | 2.993^b^ | 1.000 | 46.000 | .090 | .061 | 2.993 | .395 |
|  | Roy's Largest Root | .065 | 2.993^b^ | 1.000 | 46.000 | .090 | .061 | 2.993 | .395 |
| a. Design: Intercept + Groups  Within Subjects Design: fatige | | | | | | | | | |
| b. Exact statistic | | | | | | | | | |
| c. Computed using alpha = .05 | | | | | | | | | |

| **Tests of Within-Subjects Effects** | | | | | | | | | |
| --- | --- | --- | --- | --- | --- | --- | --- | --- | --- |
| Measure: MEASURE_1 | | | | | | | | | |
| Source | | Type III Sum of Squares | df | Mean Square | F | Sig. | Partial Eta Squared | Noncent. Parameter | Observed Power^a^ |
| fatige | Sphericity Assumed | 18866.626 | 1 | 18866.626 | 3.351 | .074 | .068 | 3.351 | .434 |
|  | Greenhouse-Geisser | 18866.626 | 1.000 | 18866.626 | 3.351 | .074 | .068 | 3.351 | .434 |
|  | Huynh-Feldt | 18866.626 | 1.000 | 18866.626 | 3.351 | .074 | .068 | 3.351 | .434 |
|  | Lower-bound | 18866.626 | 1.000 | 18866.626 | 3.351 | .074 | .068 | 3.351 | .434 |
| fatige * Groups | Sphericity Assumed | 16849.744 | 1 | 16849.744 | 2.993 | .090 | .061 | 2.993 | .395 |
|  | Greenhouse-Geisser | 16849.744 | 1.000 | 16849.744 | 2.993 | .090 | .061 | 2.993 | .395 |
|  | Huynh-Feldt | 16849.744 | 1.000 | 16849.744 | 2.993 | .090 | .061 | 2.993 | .395 |
|  | Lower-bound | 16849.744 | 1.000 | 16849.744 | 2.993 | .090 | .061 | 2.993 | .395 |
| Error(fatige) | Sphericity Assumed | 258966.523 | 46 | 5629.707 |  |  |  |  |  |
|  | Greenhouse-Geisser | 258966.523 | 46.000 | 5629.707 |  |  |  |  |  |
|  | Huynh-Feldt | 258966.523 | 46.000 | 5629.707 |  |  |  |  |  |
|  | Lower-bound | 258966.523 | 46.000 | 5629.707 |  |  |  |  |  |
| a. Computed using alpha = .05 | | | | | | | | | |

| **Tests of Within-Subjects Contrasts** | | | | | | | | | |
| --- | --- | --- | --- | --- | --- | --- | --- | --- | --- |
| Measure: MEASURE_1 | | | | | | | | | |
| Source | fatige | Type III Sum of Squares | df | Mean Square | F | Sig. | Partial Eta Squared | Noncent. Parameter | Observed Power^a^ |
| fatige | Linear | 18866.626 | 1 | 18866.626 | 3.351 | .074 | .068 | 3.351 | .434 |
| fatige * Groups | Linear | 16849.744 | 1 | 16849.744 | 2.993 | .090 | .061 | 2.993 | .395 |
| Error(fatige) | Linear | 258966.523 | 46 | 5629.707 |  |  |  |  |  |
| a. Computed using alpha = .05 | | | | | | | | | |

| **Tests of Between-Subjects Effects** | | | | | | | | |
| --- | --- | --- | --- | --- | --- | --- | --- | --- |
| Measure: MEASURE_1 | | | | | | | | |
| Transformed Variable: Average | | | | | | | | |
| Source | Type III Sum of Squares | df | Mean Square | F | Sig. | Partial Eta Squared | Noncent. Parameter | Observed Power^a^ |
| Intercept | 869099.519 | 1 | 869099.519 | 137.872 | .000 | .750 | 137.872 | 1.000 |
| Groups | 11.236 | 1 | 11.236 | .002 | .967 | .000 | .002 | .050 |
| Error | 289969.673 | 46 | 6303.689 |  |  |  |  |  |
| a. Computed using alpha = .05 | | | | | | | | |

| **Parameter Estimates** | | | | | | | | | | |
| --- | --- | --- | --- | --- | --- | --- | --- | --- | --- | --- |
| Dependent Variable | Parameter | B | Std. Error | t | Sig. | 95% Confidence Interval | | Partial Eta Squared | Noncent. Parameter | Observed Power^b^ |
|  |  |  |  |  |  | Lower Bound | Upper Bound |  |  |  |
| nVLpoPreB | Intercept | 68.223 | 11.549 | 5.907 | .000 | 44.976 | 91.469 | .431 | 5.907 | 1.000 |
|  | [Groups=1.00] | 25.812 | 16.333 | 1.580 | .121 | -7.063 | 58.688 | .052 | 1.580 | .340 |
|  | [Groups=2.00] | 0^a^ | . | . | . | . | . | . | . | . |
| nVLpoPOSB | Intercept | 122.757 | 19.075 | 6.436 | .000 | 84.362 | 161.153 | .474 | 6.436 | 1.000 |
|  | [Groups=1.00] | -27.181 | 26.976 | -1.008 | .319 | -81.481 | 27.119 | .022 | 1.008 | .167 |
|  | [Groups=2.00] | 0^a^ | . | . | . | . | . | . | . | . |
| a. This parameter is set to zero because it is redundant. | | | | | | | | | | |
| b. Computed using alpha = .05 | | | | | | | | | | |

**VM-Late stance**

| **Within-Subjects Factors** | |
| --- | --- |
| Measure: MEASURE_1 | |
| fatige | Dependent Variable |
| 1 | nVMpoPreB |
| 2 | nVMpoPOSB |

| **Between-Subjects Factors** | | | |
| --- | --- | --- | --- |
|  | | Value Label | N |
| Groups | 1.00 | noramweieght | 24 |
|  | 2.00 | over weight | 24 |

| **Descriptive Statistics** | | | | |
| --- | --- | --- | --- | --- |
|  | Groups | Mean | Std. Deviation | N |
| nVMpoPreB | noramweieght | 48.2189 | 35.20702 | 24 |
|  | over weight | 37.3660 | 16.01626 | 24 |
|  | Total | 42.7924 | 27.60769 | 48 |
| nVMpoPOSB | noramweieght | 87.0699 | 33.59247 | 24 |
|  | over weight | 89.9574 | 44.50528 | 24 |
|  | Total | 88.5137 | 39.03382 | 48 |

| **Multivariate Tests^a^** | | | | | | | | | |
| --- | --- | --- | --- | --- | --- | --- | --- | --- | --- |
| Effect | | Value | F | Hypothesis df | Error df | Sig. | Partial Eta Squared | Noncent. Parameter | Observed Power^c^ |
| fatige | Pillai's Trace | .534 | 52.761^b^ | 1.000 | 46.000 | .000 | .534 | 52.761 | 1.000 |
|  | Wilks' Lambda | .466 | 52.761^b^ | 1.000 | 46.000 | .000 | .534 | 52.761 | 1.000 |
|  | Hotelling's Trace | 1.147 | 52.761^b^ | 1.000 | 46.000 | .000 | .534 | 52.761 | 1.000 |
|  | Roy's Largest Root | 1.147 | 52.761^b^ | 1.000 | 46.000 | .000 | .534 | 52.761 | 1.000 |
| fatige * Groups | Pillai's Trace | .025 | 1.191^b^ | 1.000 | 46.000 | .281 | .025 | 1.191 | .188 |
|  | Wilks' Lambda | .975 | 1.191^b^ | 1.000 | 46.000 | .281 | .025 | 1.191 | .188 |
|  | Hotelling's Trace | .026 | 1.191^b^ | 1.000 | 46.000 | .281 | .025 | 1.191 | .188 |
|  | Roy's Largest Root | .026 | 1.191^b^ | 1.000 | 46.000 | .281 | .025 | 1.191 | .188 |
| a. Design: Intercept + Groups  Within Subjects Design: fatige | | | | | | | | | |
| b. Exact statistic | | | | | | | | | |
| c. Computed using alpha = .05 | | | | | | | | | |

| **Tests of Within-Subjects Effects** | | | | | | | | | |
| --- | --- | --- | --- | --- | --- | --- | --- | --- | --- |
| Measure: MEASURE_1 | | | | | | | | | |
| Source | | Type III Sum of Squares | df | Mean Square | F | Sig. | Partial Eta Squared | Noncent. Parameter | Observed Power^a^ |
| fatige | Sphericity Assumed | 50170.364 | 1 | 50170.364 | 52.761 | .000 | .534 | 52.761 | 1.000 |
|  | Greenhouse-Geisser | 50170.364 | 1.000 | 50170.364 | 52.761 | .000 | .534 | 52.761 | 1.000 |
|  | Huynh-Feldt | 50170.364 | 1.000 | 50170.364 | 52.761 | .000 | .534 | 52.761 | 1.000 |
|  | Lower-bound | 50170.364 | 1.000 | 50170.364 | 52.761 | .000 | .534 | 52.761 | 1.000 |
| fatige * Groups | Sphericity Assumed | 1132.787 | 1 | 1132.787 | 1.191 | .281 | .025 | 1.191 | .188 |
|  | Greenhouse-Geisser | 1132.787 | 1.000 | 1132.787 | 1.191 | .281 | .025 | 1.191 | .188 |
|  | Huynh-Feldt | 1132.787 | 1.000 | 1132.787 | 1.191 | .281 | .025 | 1.191 | .188 |
|  | Lower-bound | 1132.787 | 1.000 | 1132.787 | 1.191 | .281 | .025 | 1.191 | .188 |
| Error(fatige) | Sphericity Assumed | 43741.649 | 46 | 950.905 |  |  |  |  |  |
|  | Greenhouse-Geisser | 43741.649 | 46.000 | 950.905 |  |  |  |  |  |
|  | Huynh-Feldt | 43741.649 | 46.000 | 950.905 |  |  |  |  |  |
|  | Lower-bound | 43741.649 | 46.000 | 950.905 |  |  |  |  |  |
| a. Computed using alpha = .05 | | | | | | | | | |

| **Tests of Within-Subjects Contrasts** | | | | | | | | | |
| --- | --- | --- | --- | --- | --- | --- | --- | --- | --- |
| Measure: MEASURE_1 | | | | | | | | | |
| Source | fatige | Type III Sum of Squares | df | Mean Square | F | Sig. | Partial Eta Squared | Noncent. Parameter | Observed Power^a^ |
| fatige | Linear | 50170.364 | 1 | 50170.364 | 52.761 | .000 | .534 | 52.761 | 1.000 |
| fatige * Groups | Linear | 1132.787 | 1 | 1132.787 | 1.191 | .281 | .025 | 1.191 | .188 |
| Error(fatige) | Linear | 43741.649 | 46 | 950.905 |  |  |  |  |  |
| a. Computed using alpha = .05 | | | | | | | | | |

| **Tests of Between-Subjects Effects** | | | | | | | | |
| --- | --- | --- | --- | --- | --- | --- | --- | --- |
| Measure: MEASURE_1 | | | | | | | | |
| Transformed Variable: Average | | | | | | | | |
| Source | Type III Sum of Squares | df | Mean Square | F | Sig. | Partial Eta Squared | Noncent. Parameter | Observed Power^a^ |
| Intercept | 413791.048 | 1 | 413791.048 | 306.124 | .000 | .869 | 306.124 | 1.000 |
| Groups | 380.680 | 1 | 380.680 | .282 | .598 | .006 | .282 | .081 |
| Error | 62178.606 | 46 | 1351.709 |  |  |  |  |  |
| a. Computed using alpha = .05 | | | | | | | | |

| **Parameter Estimates** | | | | | | | | | | |
| --- | --- | --- | --- | --- | --- | --- | --- | --- | --- | --- |
| Dependent Variable | Parameter | B | Std. Error | t | Sig. | 95% Confidence Interval | | Partial Eta Squared | Noncent. Parameter | Observed Power^b^ |
|  |  |  |  |  |  | Lower Bound | Upper Bound |  |  |  |
| nVMpoPreB | Intercept | 37.366 | 5.583 | 6.693 | .000 | 26.128 | 48.604 | .493 | 6.693 | 1.000 |
|  | [Groups=1.00] | 10.853 | 7.895 | 1.375 | .176 | -5.040 | 26.745 | .039 | 1.375 | .270 |
|  | [Groups=2.00] | 0^a^ | . | . | . | . | . | . | . | . |
| nVMpoPOSB | Intercept | 89.957 | 8.048 | 11.177 | .000 | 73.757 | 106.158 | .731 | 11.177 | 1.000 |
|  | [Groups=1.00] | -2.888 | 11.382 | -.254 | .801 | -25.798 | 20.023 | .001 | .254 | .057 |
|  | [Groups=2.00] | 0^a^ | . | . | . | . | . | . | . | . |
| a. This parameter is set to zero because it is redundant. | | | | | | | | | | |
| b. Computed using alpha = .05 | | | | | | | | | | |

**RF- Late stance**

| **Within-Subjects Factors** | |
| --- | --- |
| Measure: MEASURE_1 | |
| fatige | Dependent Variable |
| 1 | nRFpoPreB |
| 2 | nRFpoPOSB |

| **Between-Subjects Factors** | | | |
| --- | --- | --- | --- |
|  | | Value Label | N |
| Groups | 1.00 | noramweieght | 24 |
|  | 2.00 | over weight | 24 |

| **Descriptive Statistics** | | | | |
| --- | --- | --- | --- | --- |
|  | Groups | Mean | Std. Deviation | N |
| nRFpoPreB | noramweieght | 44.8619 | 30.34095 | 24 |
|  | over weight | 71.7532 | 44.63831 | 24 |
|  | Total | 58.3075 | 40.12750 | 48 |
| nRFpoPOSB | noramweieght | 56.0996 | 40.91029 | 24 |
|  | over weight | 86.8240 | 60.41385 | 24 |
|  | Total | 71.4618 | 53.34912 | 48 |

| **Multivariate Tests^a^** | | | | | | | | | |
| --- | --- | --- | --- | --- | --- | --- | --- | --- | --- |
| Effect | | Value | F | Hypothesis df | Error df | Sig. | Partial Eta Squared | Noncent. Parameter | Observed Power^c^ |
| fatige | Pillai's Trace | .197 | 11.299^b^ | 1.000 | 46.000 | .002 | .197 | 11.299 | .908 |
|  | Wilks' Lambda | .803 | 11.299^b^ | 1.000 | 46.000 | .002 | .197 | 11.299 | .908 |
|  | Hotelling's Trace | .246 | 11.299^b^ | 1.000 | 46.000 | .002 | .197 | 11.299 | .908 |
|  | Roy's Largest Root | .246 | 11.299^b^ | 1.000 | 46.000 | .002 | .197 | 11.299 | .908 |
| fatige * Groups | Pillai's Trace | .005 | .240^b^ | 1.000 | 46.000 | .627 | .005 | .240 | .077 |
|  | Wilks' Lambda | .995 | .240^b^ | 1.000 | 46.000 | .627 | .005 | .240 | .077 |
|  | Hotelling's Trace | .005 | .240^b^ | 1.000 | 46.000 | .627 | .005 | .240 | .077 |
|  | Roy's Largest Root | .005 | .240^b^ | 1.000 | 46.000 | .627 | .005 | .240 | .077 |
| a. Design: Intercept + Groups  Within Subjects Design: fatige | | | | | | | | | |
| b. Exact statistic | | | | | | | | | |
| c. Computed using alpha = .05 | | | | | | | | | |

| **Tests of Within-Subjects Effects** | | | | | | | | | |
| --- | --- | --- | --- | --- | --- | --- | --- | --- | --- |
| Measure: MEASURE_1 | | | | | | | | | |
| Source | | Type III Sum of Squares | df | Mean Square | F | Sig. | Partial Eta Squared | Noncent. Parameter | Observed Power^a^ |
| fatige | Sphericity Assumed | 4152.860 | 1 | 4152.860 | 11.299 | .002 | .197 | 11.299 | .908 |
|  | Greenhouse-Geisser | 4152.860 | 1.000 | 4152.860 | 11.299 | .002 | .197 | 11.299 | .908 |
|  | Huynh-Feldt | 4152.860 | 1.000 | 4152.860 | 11.299 | .002 | .197 | 11.299 | .908 |
|  | Lower-bound | 4152.860 | 1.000 | 4152.860 | 11.299 | .002 | .197 | 11.299 | .908 |
| fatige * Groups | Sphericity Assumed | 88.157 | 1 | 88.157 | .240 | .627 | .005 | .240 | .077 |
|  | Greenhouse-Geisser | 88.157 | 1.000 | 88.157 | .240 | .627 | .005 | .240 | .077 |
|  | Huynh-Feldt | 88.157 | 1.000 | 88.157 | .240 | .627 | .005 | .240 | .077 |
|  | Lower-bound | 88.157 | 1.000 | 88.157 | .240 | .627 | .005 | .240 | .077 |
| Error(fatige) | Sphericity Assumed | 16906.957 | 46 | 367.543 |  |  |  |  |  |
|  | Greenhouse-Geisser | 16906.957 | 46.000 | 367.543 |  |  |  |  |  |
|  | Huynh-Feldt | 16906.957 | 46.000 | 367.543 |  |  |  |  |  |
|  | Lower-bound | 16906.957 | 46.000 | 367.543 |  |  |  |  |  |
| a. Computed using alpha = .05 | | | | | | | | | |

| **Tests of Within-Subjects Contrasts** | | | | | | | | | |
| --- | --- | --- | --- | --- | --- | --- | --- | --- | --- |
| Measure: MEASURE_1 | | | | | | | | | |
| Source | fatige | Type III Sum of Squares | df | Mean Square | F | Sig. | Partial Eta Squared | Noncent. Parameter | Observed Power^a^ |
| fatige | Linear | 4152.860 | 1 | 4152.860 | 11.299 | .002 | .197 | 11.299 | .908 |
| fatige * Groups | Linear | 88.157 | 1 | 88.157 | .240 | .627 | .005 | .240 | .077 |
| Error(fatige) | Linear | 16906.957 | 46 | 367.543 |  |  |  |  |  |
| a. Computed using alpha = .05 | | | | | | | | | |

| **Tests of Between-Subjects Effects** | | | | | | | | |
| --- | --- | --- | --- | --- | --- | --- | --- | --- |
| Measure: MEASURE_1 | | | | | | | | |
| Transformed Variable: Average | | | | | | | | |
| Source | Type III Sum of Squares | df | Mean Square | F | Sig. | Partial Eta Squared | Noncent. Parameter | Observed Power^a^ |
| Intercept | 404162.006 | 1 | 404162.006 | 107.754 | .000 | .701 | 107.754 | 1.000 |
| Groups | 19917.402 | 1 | 19917.402 | 5.310 | .026 | .103 | 5.310 | .616 |
| Error | 172535.676 | 46 | 3750.776 |  |  |  |  |  |
| a. Computed using alpha = .05 | | | | | | | | |

| **Parameter Estimates** | | | | | | | | | | |
| --- | --- | --- | --- | --- | --- | --- | --- | --- | --- | --- |
| Dependent Variable | Parameter | B | Std. Error | t | Sig. | 95% Confidence Interval | | Partial Eta Squared | Noncent. Parameter | Observed Power^b^ |
|  |  |  |  |  |  | Lower Bound | Upper Bound |  |  |  |
| nRFpoPreB | Intercept | 71.753 | 7.790 | 9.210 | .000 | 56.072 | 87.434 | .648 | 9.210 | 1.000 |
|  | [Groups=1.00] | -26.891 | 11.017 | -2.441 | .019 | -49.068 | -4.715 | .115 | 2.441 | .666 |
|  | [Groups=2.00] | 0^a^ | . | . | . | . | . | . | . | . |
| nRFpoPOSB | Intercept | 86.824 | 10.531 | 8.244 | .000 | 65.626 | 108.022 | .596 | 8.244 | 1.000 |
|  | [Groups=1.00] | -30.724 | 14.893 | -2.063 | .045 | -60.703 | -.746 | .085 | 2.063 | .524 |
|  | [Groups=2.00] | 0^a^ | . | . | . | . | . | . | . | . |
| a. This parameter is set to zero because it is redundant. | | | | | | | | | | |
| b. Computed using alpha = .05 | | | | | | | | | | |

**BF-Late stance**

| **Within-Subjects Factors** | |
| --- | --- |
| Measure: MEASURE_1 | |
| fatige | Dependent Variable |
| 1 | nBFpoPreB |
| 2 | nBFpoPOSB |

| **Between-Subjects Factors** | | | |
| --- | --- | --- | --- |
|  | | Value Label | N |
| Groups | 1.00 | noramweieght | 24 |
|  | 2.00 | over weight | 24 |

| **Descriptive Statistics** | | | | |
| --- | --- | --- | --- | --- |
|  | Groups | Mean | Std. Deviation | N |
| nBFpoPreB | noramweieght | 37.6697 | 17.43163 | 24 |
|  | over weight | 47.7684 | 29.09510 | 24 |
|  | Total | 42.7190 | 24.26919 | 48 |
| nBFpoPOSB | noramweieght | 52.7424 | 42.88338 | 24 |
|  | over weight | 48.4134 | 21.30389 | 24 |
|  | Total | 50.5779 | 33.56804 | 48 |

| **Multivariate Tests^a^** | | | | | | | | | |
| --- | --- | --- | --- | --- | --- | --- | --- | --- | --- |
| Effect | | Value | F | Hypothesis df | Error df | Sig. | Partial Eta Squared | Noncent. Parameter | Observed Power^c^ |
| fatige | Pillai's Trace | .054 | 2.609^b^ | 1.000 | 46.000 | .113 | .054 | 2.609 | .353 |
|  | Wilks' Lambda | .946 | 2.609^b^ | 1.000 | 46.000 | .113 | .054 | 2.609 | .353 |
|  | Hotelling's Trace | .057 | 2.609^b^ | 1.000 | 46.000 | .113 | .054 | 2.609 | .353 |
|  | Roy's Largest Root | .057 | 2.609^b^ | 1.000 | 46.000 | .113 | .054 | 2.609 | .353 |
| fatige * Groups | Pillai's Trace | .046 | 2.198^b^ | 1.000 | 46.000 | .145 | .046 | 2.198 | .306 |
|  | Wilks' Lambda | .954 | 2.198^b^ | 1.000 | 46.000 | .145 | .046 | 2.198 | .306 |
|  | Hotelling's Trace | .048 | 2.198^b^ | 1.000 | 46.000 | .145 | .046 | 2.198 | .306 |
|  | Roy's Largest Root | .048 | 2.198^b^ | 1.000 | 46.000 | .145 | .046 | 2.198 | .306 |
| a. Design: Intercept + Groups  Within Subjects Design: fatige | | | | | | | | | |
| b. Exact statistic | | | | | | | | | |
| c. Computed using alpha = .05 | | | | | | | | | |

| **Tests of Within-Subjects Effects** | | | | | | | | | |
| --- | --- | --- | --- | --- | --- | --- | --- | --- | --- |
| Measure: MEASURE_1 | | | | | | | | | |
| Source | | Type III Sum of Squares | df | Mean Square | F | Sig. | Partial Eta Squared | Noncent. Parameter | Observed Power^a^ |
| fatige | Sphericity Assumed | 1482.278 | 1 | 1482.278 | 2.609 | .113 | .054 | 2.609 | .353 |
|  | Greenhouse-Geisser | 1482.278 | 1.000 | 1482.278 | 2.609 | .113 | .054 | 2.609 | .353 |
|  | Huynh-Feldt | 1482.278 | 1.000 | 1482.278 | 2.609 | .113 | .054 | 2.609 | .353 |
|  | Lower-bound | 1482.278 | 1.000 | 1482.278 | 2.609 | .113 | .054 | 2.609 | .353 |
| fatige * Groups | Sphericity Assumed | 1248.959 | 1 | 1248.959 | 2.198 | .145 | .046 | 2.198 | .306 |
|  | Greenhouse-Geisser | 1248.959 | 1.000 | 1248.959 | 2.198 | .145 | .046 | 2.198 | .306 |
|  | Huynh-Feldt | 1248.959 | 1.000 | 1248.959 | 2.198 | .145 | .046 | 2.198 | .306 |
|  | Lower-bound | 1248.959 | 1.000 | 1248.959 | 2.198 | .145 | .046 | 2.198 | .306 |
| Error(fatige) | Sphericity Assumed | 26135.810 | 46 | 568.170 |  |  |  |  |  |
|  | Greenhouse-Geisser | 26135.810 | 46.000 | 568.170 |  |  |  |  |  |
|  | Huynh-Feldt | 26135.810 | 46.000 | 568.170 |  |  |  |  |  |
|  | Lower-bound | 26135.810 | 46.000 | 568.170 |  |  |  |  |  |
| a. Computed using alpha = .05 | | | | | | | | | |

| **Tests of Within-Subjects Contrasts** | | | | | | | | | |
| --- | --- | --- | --- | --- | --- | --- | --- | --- | --- |
| Measure: MEASURE_1 | | | | | | | | | |
| Source | fatige | Type III Sum of Squares | df | Mean Square | F | Sig. | Partial Eta Squared | Noncent. Parameter | Observed Power^a^ |
| fatige | Linear | 1482.278 | 1 | 1482.278 | 2.609 | .113 | .054 | 2.609 | .353 |
| fatige * Groups | Linear | 1248.959 | 1 | 1248.959 | 2.198 | .145 | .046 | 2.198 | .306 |
| Error(fatige) | Linear | 26135.810 | 46 | 568.170 |  |  |  |  |  |
| a. Computed using alpha = .05 | | | | | | | | | |

| **Tests of Between-Subjects Effects** | | | | | | | | |
| --- | --- | --- | --- | --- | --- | --- | --- | --- |
| Measure: MEASURE_1 | | | | | | | | |
| Transformed Variable: Average | | | | | | | | |
| Source | Type III Sum of Squares | df | Mean Square | F | Sig. | Partial Eta Squared | Noncent. Parameter | Observed Power^a^ |
| Intercept | 208903.579 | 1 | 208903.579 | 181.113 | .000 | .797 | 181.113 | 1.000 |
| Groups | 199.731 | 1 | 199.731 | .173 | .679 | .004 | .173 | .069 |
| Error | 53058.419 | 46 | 1153.444 |  |  |  |  |  |
| a. Computed using alpha = .05 | | | | | | | | |

| **Parameter Estimates** | | | | | | | | | | |
| --- | --- | --- | --- | --- | --- | --- | --- | --- | --- | --- |
| Dependent Variable | Parameter | B | Std. Error | t | Sig. | 95% Confidence Interval | | Partial Eta Squared | Noncent. Parameter | Observed Power^b^ |
|  |  |  |  |  |  | Lower Bound | Upper Bound |  |  |  |
| nBFpoPreB | Intercept | 47.768 | 4.896 | 9.758 | .000 | 37.914 | 57.623 | .674 | 9.758 | 1.000 |
|  | [Groups=1.00] | -10.099 | 6.923 | -1.459 | .151 | -24.035 | 3.837 | .044 | 1.459 | .298 |
|  | [Groups=2.00] | 0^a^ | . | . | . | . | . | . | . | . |
| nBFpoPOSB | Intercept | 48.413 | 6.911 | 7.005 | .000 | 34.501 | 62.325 | .516 | 7.005 | 1.000 |
|  | [Groups=1.00] | 4.329 | 9.774 | .443 | .660 | -15.345 | 24.004 | .004 | .443 | .072 |
|  | [Groups=2.00] | 0^a^ | . | . | . | . | . | . | . | . |
| a. This parameter is set to zero because it is redundant. | | | | | | | | | | |
| b. Computed using alpha = .05 | | | | | | | | | | |

**ST-Late stance**

| **Within-Subjects Factors** | |
| --- | --- |
| Measure: MEASURE_1 | |
| fatige | Dependent Variable |
| 1 | nSTpoPreB |
| 2 | nSTpoPOSB |

| **Between-Subjects Factors** | | | |
| --- | --- | --- | --- |
|  | | Value Label | N |
| Groups | 1.00 | noramweieght | 24 |
|  | 2.00 | over weight | 24 |

| **Descriptive Statistics** | | | | |
| --- | --- | --- | --- | --- |
|  | Groups | Mean | Std. Deviation | N |
| nSTpoPreB | noramweieght | 42.6353 | 19.02309 | 24 |
|  | over weight | 53.5140 | 28.56090 | 24 |
|  | Total | 48.0747 | 24.62702 | 48 |
| nSTpoPOSB | noramweieght | 70.9077 | 58.40460 | 24 |
|  | over weight | 69.5839 | 48.65947 | 24 |
|  | Total | 70.2458 | 53.18262 | 48 |

| **Multivariate Tests^a^** | | | | | | | | | |
| --- | --- | --- | --- | --- | --- | --- | --- | --- | --- |
| Effect | | Value | F | Hypothesis df | Error df | Sig. | Partial Eta Squared | Noncent. Parameter | Observed Power^c^ |
| fatige | Pillai's Trace | .133 | 7.039^b^ | 1.000 | 46.000 | .011 | .133 | 7.039 | .738 |
|  | Wilks' Lambda | .867 | 7.039^b^ | 1.000 | 46.000 | .011 | .133 | 7.039 | .738 |
|  | Hotelling's Trace | .153 | 7.039^b^ | 1.000 | 46.000 | .011 | .133 | 7.039 | .738 |
|  | Roy's Largest Root | .153 | 7.039^b^ | 1.000 | 46.000 | .011 | .133 | 7.039 | .738 |
| fatige * Groups | Pillai's Trace | .011 | .533^b^ | 1.000 | 46.000 | .469 | .011 | .533 | .110 |
|  | Wilks' Lambda | .989 | .533^b^ | 1.000 | 46.000 | .469 | .011 | .533 | .110 |
|  | Hotelling's Trace | .012 | .533^b^ | 1.000 | 46.000 | .469 | .011 | .533 | .110 |
|  | Roy's Largest Root | .012 | .533^b^ | 1.000 | 46.000 | .469 | .011 | .533 | .110 |
| a. Design: Intercept + Groups  Within Subjects Design: fatige | | | | | | | | | |
| b. Exact statistic | | | | | | | | | |
| c. Computed using alpha = .05 | | | | | | | | | |

| **Tests of Within-Subjects Effects** | | | | | | | | | |
| --- | --- | --- | --- | --- | --- | --- | --- | --- | --- |
| Measure: MEASURE_1 | | | | | | | | | |
| Source | | Type III Sum of Squares | df | Mean Square | F | Sig. | Partial Eta Squared | Noncent. Parameter | Observed Power^a^ |
| fatige | Sphericity Assumed | 11797.402 | 1 | 11797.402 | 7.039 | .011 | .133 | 7.039 | .738 |
|  | Greenhouse-Geisser | 11797.402 | 1.000 | 11797.402 | 7.039 | .011 | .133 | 7.039 | .738 |
|  | Huynh-Feldt | 11797.402 | 1.000 | 11797.402 | 7.039 | .011 | .133 | 7.039 | .738 |
|  | Lower-bound | 11797.402 | 1.000 | 11797.402 | 7.039 | .011 | .133 | 7.039 | .738 |
| fatige * Groups | Sphericity Assumed | 893.407 | 1 | 893.407 | .533 | .469 | .011 | .533 | .110 |
|  | Greenhouse-Geisser | 893.407 | 1.000 | 893.407 | .533 | .469 | .011 | .533 | .110 |
|  | Huynh-Feldt | 893.407 | 1.000 | 893.407 | .533 | .469 | .011 | .533 | .110 |
|  | Lower-bound | 893.407 | 1.000 | 893.407 | .533 | .469 | .011 | .533 | .110 |
| Error(fatige) | Sphericity Assumed | 77098.307 | 46 | 1676.050 |  |  |  |  |  |
|  | Greenhouse-Geisser | 77098.307 | 46.000 | 1676.050 |  |  |  |  |  |
|  | Huynh-Feldt | 77098.307 | 46.000 | 1676.050 |  |  |  |  |  |
|  | Lower-bound | 77098.307 | 46.000 | 1676.050 |  |  |  |  |  |
| a. Computed using alpha = .05 | | | | | | | | | |

| **Tests of Within-Subjects Contrasts** | | | | | | | | | |
| --- | --- | --- | --- | --- | --- | --- | --- | --- | --- |
| Measure: MEASURE_1 | | | | | | | | | |
| Source | fatige | Type III Sum of Squares | df | Mean Square | F | Sig. | Partial Eta Squared | Noncent. Parameter | Observed Power^a^ |
| fatige | Linear | 11797.402 | 1 | 11797.402 | 7.039 | .011 | .133 | 7.039 | .738 |
| fatige * Groups | Linear | 893.407 | 1 | 893.407 | .533 | .469 | .011 | .533 | .110 |
| Error(fatige) | Linear | 77098.307 | 46 | 1676.050 |  |  |  |  |  |
| a. Computed using alpha = .05 | | | | | | | | | |

| **Tests of Between-Subjects Effects** | | | | | | | | |
| --- | --- | --- | --- | --- | --- | --- | --- | --- |
| Measure: MEASURE_1 | | | | | | | | |
| Transformed Variable: Average | | | | | | | | |
| Source | Type III Sum of Squares | df | Mean Square | F | Sig. | Partial Eta Squared | Noncent. Parameter | Observed Power^a^ |
| Intercept | 335993.582 | 1 | 335993.582 | 186.438 | .000 | .802 | 186.438 | 1.000 |
| Groups | 547.785 | 1 | 547.785 | .304 | .584 | .007 | .304 | .084 |
| Error | 82899.910 | 46 | 1802.172 |  |  |  |  |  |
| a. Computed using alpha = .05 | | | | | | | | |

| **Parameter Estimates** | | | | | | | | | | |
| --- | --- | --- | --- | --- | --- | --- | --- | --- | --- | --- |
| Dependent Variable | Parameter | B | Std. Error | t | Sig. | 95% Confidence Interval | | Partial Eta Squared | Noncent. Parameter | Observed Power^b^ |
|  |  |  |  |  |  | Lower Bound | Upper Bound |  |  |  |
| nSTpoPreB | Intercept | 53.514 | 4.953 | 10.804 | .000 | 43.544 | 63.484 | .717 | 10.804 | 1.000 |
|  | [Groups=1.00] | -10.879 | 7.005 | -1.553 | .127 | -24.979 | 3.221 | .050 | 1.553 | .330 |
|  | [Groups=2.00] | 0^a^ | . | . | . | . | . | . | . | . |
| nSTpoPOSB | Intercept | 69.584 | 10.972 | 6.342 | .000 | 47.498 | 91.670 | .466 | 6.342 | 1.000 |
|  | [Groups=1.00] | 1.324 | 15.517 | .085 | .932 | -29.911 | 32.558 | .000 | .085 | .051 |
|  | [Groups=2.00] | 0^a^ | . | . | . | . | . | . | . | . |
| a. This parameter is set to zero because it is redundant. | | | | | | | | | | |
| b. Computed using alpha = .05 | | | | | | | | | | |

**General knee co-contraction**

| **Within-Subjects Factors** | |
| --- | --- |
| Measure: MEASURE_1 | |
| factor1 | Dependent Variable |
| 1 | generalCokneePreA |
| 2 | generalCoKneePosA |

| **Between-Subjects Factors** | | | |
| --- | --- | --- | --- |
|  | | Value Label | N |
| Groups | 1.00 | noramweieght | 24 |
|  | 2.00 | over weight | 24 |

| **Descriptive Statistics** | | | | |
| --- | --- | --- | --- | --- |
|  | Groups | Mean | Std. Deviation | N |
| generalCokneePreA | noramweieght | 205.8290 | 76.78800 | 24 |
|  | over weight | 224.2929 | 106.48290 | 24 |
|  | Total | 215.0609 | 92.31031 | 48 |
| generalCoKneePosA | noramweieght | 276.1556 | 139.46089 | 24 |
|  | over weight | 321.0209 | 171.33816 | 24 |
|  | Total | 298.5882 | 156.19785 | 48 |

| **Multivariate Tests^a^** | | | | | | | | | |
| --- | --- | --- | --- | --- | --- | --- | --- | --- | --- |
| Effect | | Value | F | Hypothesis df | Error df | Sig. | Partial Eta Squared | Noncent. Parameter | Observed Power^c^ |
| factor1 | Pillai's Trace | .244 | 14.864^b^ | 1.000 | 46.000 | .000 | .244 | 14.864 | .965 |
|  | Wilks' Lambda | .756 | 14.864^b^ | 1.000 | 46.000 | .000 | .244 | 14.864 | .965 |
|  | Hotelling's Trace | .323 | 14.864^b^ | 1.000 | 46.000 | .000 | .244 | 14.864 | .965 |
|  | Roy's Largest Root | .323 | 14.864^b^ | 1.000 | 46.000 | .000 | .244 | 14.864 | .965 |
| factor1 * Groups | Pillai's Trace | .008 | .371^b^ | 1.000 | 46.000 | .545 | .008 | .371 | .092 |
|  | Wilks' Lambda | .992 | .371^b^ | 1.000 | 46.000 | .545 | .008 | .371 | .092 |
|  | Hotelling's Trace | .008 | .371^b^ | 1.000 | 46.000 | .545 | .008 | .371 | .092 |
|  | Roy's Largest Root | .008 | .371^b^ | 1.000 | 46.000 | .545 | .008 | .371 | .092 |
| a. Design: Intercept + Groups  Within Subjects Design: factor1 | | | | | | | | | |
| b. Exact statistic | | | | | | | | | |
| c. Computed using alpha = .05 | | | | | | | | | |

| **Tests of Within-Subjects Effects** | | | | | | | | | |
| --- | --- | --- | --- | --- | --- | --- | --- | --- | --- |
| Measure: MEASURE_1 | | | | | | | | | |
| Source | | Type III Sum of Squares | df | Mean Square | F | Sig. | Partial Eta Squared | Noncent. Parameter | Observed Power^a^ |
| factor1 | Sphericity Assumed | 167443.559 | 1 | 167443.559 | 14.864 | .000 | .244 | 14.864 | .965 |
|  | Greenhouse-Geisser | 167443.559 | 1.000 | 167443.559 | 14.864 | .000 | .244 | 14.864 | .965 |
|  | Huynh-Feldt | 167443.559 | 1.000 | 167443.559 | 14.864 | .000 | .244 | 14.864 | .965 |
|  | Lower-bound | 167443.559 | 1.000 | 167443.559 | 14.864 | .000 | .244 | 14.864 | .965 |
| factor1 * Groups | Sphericity Assumed | 4182.205 | 1 | 4182.205 | .371 | .545 | .008 | .371 | .092 |
|  | Greenhouse-Geisser | 4182.205 | 1.000 | 4182.205 | .371 | .545 | .008 | .371 | .092 |
|  | Huynh-Feldt | 4182.205 | 1.000 | 4182.205 | .371 | .545 | .008 | .371 | .092 |
|  | Lower-bound | 4182.205 | 1.000 | 4182.205 | .371 | .545 | .008 | .371 | .092 |
| Error(factor1) | Sphericity Assumed | 518200.052 | 46 | 11265.219 |  |  |  |  |  |
|  | Greenhouse-Geisser | 518200.052 | 46.000 | 11265.219 |  |  |  |  |  |
|  | Huynh-Feldt | 518200.052 | 46.000 | 11265.219 |  |  |  |  |  |
|  | Lower-bound | 518200.052 | 46.000 | 11265.219 |  |  |  |  |  |
| a. Computed using alpha = .05 | | | | | | | | | |

| **Tests of Within-Subjects Contrasts** | | | | | | | | | |
| --- | --- | --- | --- | --- | --- | --- | --- | --- | --- |
| Measure: MEASURE_1 | | | | | | | | | |
| Source | factor1 | Type III Sum of Squares | df | Mean Square | F | Sig. | Partial Eta Squared | Noncent. Parameter | Observed Power^a^ |
| factor1 | Linear | 167443.559 | 1 | 167443.559 | 14.864 | .000 | .244 | 14.864 | .965 |
| factor1 * Groups | Linear | 4182.205 | 1 | 4182.205 | .371 | .545 | .008 | .371 | .092 |
| Error(factor1) | Linear | 518200.052 | 46 | 11265.219 |  |  |  |  |  |
| a. Computed using alpha = .05 | | | | | | | | | |

| **Tests of Between-Subjects Effects** | | | | | | | | |
| --- | --- | --- | --- | --- | --- | --- | --- | --- |
| Measure: MEASURE_1 | | | | | | | | |
| Transformed Variable: Average | | | | | | | | |
| Source | Type III Sum of Squares | df | Mean Square | F | Sig. | Partial Eta Squared | Noncent. Parameter | Observed Power^a^ |
| Intercept | 6332051.133 | 1 | 6332051.133 | 291.057 | .000 | .864 | 291.057 | 1.000 |
| Groups | 24063.566 | 1 | 24063.566 | 1.106 | .298 | .023 | 1.106 | .178 |
| Error | 1000745.402 | 46 | 21755.335 |  |  |  |  |  |
| a. Computed using alpha = .05 | | | | | | | | |

| **Parameter Estimates** | | | | | | | | | | |
| --- | --- | --- | --- | --- | --- | --- | --- | --- | --- | --- |
| Dependent Variable | Parameter | B | Std. Error | t | Sig. | 95% Confidence Interval | | Partial Eta Squared | Noncent. Parameter | Observed Power^b^ |
|  |  |  |  |  |  | Lower Bound | Upper Bound |  |  |  |
| generalCokneePreA | Intercept | 224.293 | 18.949 | 11.837 | .000 | 186.151 | 262.435 | .753 | 11.837 | 1.000 |
|  | [Groups=1.00] | -18.464 | 26.798 | -.689 | .494 | -72.405 | 35.477 | .010 | .689 | .104 |
|  | [Groups=2.00] | 0^a^ | . | . | . | . | . | . | . | . |
| generalCoKneePosA | Intercept | 321.021 | 31.887 | 10.067 | .000 | 256.835 | 385.207 | .688 | 10.067 | 1.000 |
|  | [Groups=1.00] | -44.865 | 45.095 | -.995 | .325 | -135.637 | 45.907 | .021 | .995 | .164 |
|  | [Groups=2.00] | 0^a^ | . | . | . | . | . | . | . | . |
| a. This parameter is set to zero because it is redundant. | | | | | | | | | | |
| b. Computed using alpha = .05 | | | | | | | | | | |

**Directed**

**knee Flexor/Extensor co-contraction- Early stance**

| **Within-Subjects Factors** | |
| --- | --- |
| Measure: MEASURE_1 | |
| factor1 | Dependent Variable |
| 1 | flexexkneeAPRE |
| 2 | flexexkneeAPOS |

| **Between-Subjects Factors** | | | |
| --- | --- | --- | --- |
|  | | Value Label | N |
| Groups | 1.00 | noramweieght | 24 |
|  | 2.00 | over weight | 24 |

| **Descriptive Statistics** | | | | |
| --- | --- | --- | --- | --- |
|  | Groups | Mean | Std. Deviation | N |
| flexexkneeAPRE | noramweieght | .3795 | .42337 | 24 |
|  | over weight | .2114 | .50339 | 24 |
|  | Total | .2954 | .46791 | 48 |
| flexexkneeAPOS | noramweieght | .0121 | .81832 | 24 |
|  | over weight | .0868 | .57118 | 24 |
|  | Total | .0494 | .69913 | 48 |

| **Multivariate Tests^a^** | | | | | | | | | |
| --- | --- | --- | --- | --- | --- | --- | --- | --- | --- |
| Effect | | Value | F | Hypothesis df | Error df | Sig. | Partial Eta Squared | Noncent. Parameter | Observed Power^c^ |
| factor1 | Pillai's Trace | .099 | 5.037^b^ | 1.000 | 46.000 | .030 | .099 | 5.037 | .594 |
|  | Wilks' Lambda | .901 | 5.037^b^ | 1.000 | 46.000 | .030 | .099 | 5.037 | .594 |
|  | Hotelling's Trace | .110 | 5.037^b^ | 1.000 | 46.000 | .030 | .099 | 5.037 | .594 |
|  | Roy's Largest Root | .110 | 5.037^b^ | 1.000 | 46.000 | .030 | .099 | 5.037 | .594 |
| factor1 * Groups | Pillai's Trace | .026 | 1.227^b^ | 1.000 | 46.000 | .274 | .026 | 1.227 | .192 |
|  | Wilks' Lambda | .974 | 1.227^b^ | 1.000 | 46.000 | .274 | .026 | 1.227 | .192 |
|  | Hotelling's Trace | .027 | 1.227^b^ | 1.000 | 46.000 | .274 | .026 | 1.227 | .192 |
|  | Roy's Largest Root | .027 | 1.227^b^ | 1.000 | 46.000 | .274 | .026 | 1.227 | .192 |
| a. Design: Intercept + Groups  Within Subjects Design: factor1 | | | | | | | | | |
| b. Exact statistic | | | | | | | | | |
| c. Computed using alpha = .05 | | | | | | | | | |

| **Tests of Within-Subjects Effects** | | | | | | | | | |
| --- | --- | --- | --- | --- | --- | --- | --- | --- | --- |
| Measure: MEASURE_1 | | | | | | | | | |
| Source | | Type III Sum of Squares | df | Mean Square | F | Sig. | Partial Eta Squared | Noncent. Parameter | Observed Power^a^ |
| factor1 | Sphericity Assumed | 1.452 | 1 | 1.452 | 5.037 | .030 | .099 | 5.037 | .594 |
|  | Greenhouse-Geisser | 1.452 | 1.000 | 1.452 | 5.037 | .030 | .099 | 5.037 | .594 |
|  | Huynh-Feldt | 1.452 | 1.000 | 1.452 | 5.037 | .030 | .099 | 5.037 | .594 |
|  | Lower-bound | 1.452 | 1.000 | 1.452 | 5.037 | .030 | .099 | 5.037 | .594 |
| factor1 * Groups | Sphericity Assumed | .354 | 1 | .354 | 1.227 | .274 | .026 | 1.227 | .192 |
|  | Greenhouse-Geisser | .354 | 1.000 | .354 | 1.227 | .274 | .026 | 1.227 | .192 |
|  | Huynh-Feldt | .354 | 1.000 | .354 | 1.227 | .274 | .026 | 1.227 | .192 |
|  | Lower-bound | .354 | 1.000 | .354 | 1.227 | .274 | .026 | 1.227 | .192 |
| Error(factor1) | Sphericity Assumed | 13.263 | 46 | .288 |  |  |  |  |  |
|  | Greenhouse-Geisser | 13.263 | 46.000 | .288 |  |  |  |  |  |
|  | Huynh-Feldt | 13.263 | 46.000 | .288 |  |  |  |  |  |
|  | Lower-bound | 13.263 | 46.000 | .288 |  |  |  |  |  |
| a. Computed using alpha = .05 | | | | | | | | | |

| **Tests of Within-Subjects Contrasts** | | | | | | | | | |
| --- | --- | --- | --- | --- | --- | --- | --- | --- | --- |
| Measure: MEASURE_1 | | | | | | | | | |
| Source | factor1 | Type III Sum of Squares | df | Mean Square | F | Sig. | Partial Eta Squared | Noncent. Parameter | Observed Power^a^ |
| factor1 | Linear | 1.452 | 1 | 1.452 | 5.037 | .030 | .099 | 5.037 | .594 |
| factor1 * Groups | Linear | .354 | 1 | .354 | 1.227 | .274 | .026 | 1.227 | .192 |
| Error(factor1) | Linear | 13.263 | 46 | .288 |  |  |  |  |  |
| a. Computed using alpha = .05 | | | | | | | | | |

|  |
| --- |

| **Tests of Between-Subjects Effects** | | | | | | | | |
| --- | --- | --- | --- | --- | --- | --- | --- | --- |
| Measure: MEASURE_1 | | | | | | | | |
| Transformed Variable: Average | | | | | | | | |
| Source | Type III Sum of Squares | df | Mean Square | F | Sig. | Partial Eta Squared | Noncent. Parameter | Observed Power^a^ |
| Intercept | 2.855 | 1 | 2.855 | 6.702 | .013 | .127 | 6.702 | .717 |
| Groups | .052 | 1 | .052 | .123 | .727 | .003 | .123 | .064 |
| Error | 19.594 | 46 | .426 |  |  |  |  |  |
| a. Computed using alpha = .05 | | | | | | | | |

| **Parameter Estimates** | | | | | | | | | | |
| --- | --- | --- | --- | --- | --- | --- | --- | --- | --- | --- |
| Dependent Variable | Parameter | B | Std. Error | t | Sig. | 95% Confidence Interval | | Partial Eta Squared | Noncent. Parameter | Observed Power^b^ |
|  |  |  |  |  |  | Lower Bound | Upper Bound |  |  |  |
| flexexkneeAPRE | Intercept | .211 | .095 | 2.227 | .031 | .020 | .402 | .097 | 2.227 | .587 |
|  | [Groups=1.00] | .168 | .134 | 1.252 | .217 | -.102 | .438 | .033 | 1.252 | .232 |
|  | [Groups=2.00] | 0^a^ | . | . | . | . | . | . | . | . |
| flexexkneeAPOS | Intercept | .087 | .144 | .603 | .550 | -.203 | .377 | .008 | .603 | .091 |
|  | [Groups=1.00] | -.075 | .204 | -.367 | .716 | -.485 | .335 | .003 | .367 | .065 |
|  | [Groups=2.00] | 0^a^ | . | . | . | . | . | . | . | . |
| a. This parameter is set to zero because it is redundant. | | | | | | | | | | |
| b. Computed using alpha = .05 | | | | | | | | | | |

**Directed knee medio/lateral co-contraction –Early stance**

| **Within-Subjects Factors** | |
| --- | --- |
| Measure: MEASURE_1 | |
| factor1 | Dependent Variable |
| 1 | MLkneeAPRE |
| 2 | MLkneeAPOS |

| **Between-Subjects Factors** | | | |
| --- | --- | --- | --- |
|  | | Value Label | N |
| Groups | 1.00 | noramweieght | 24 |
|  | 2.00 | over weight | 24 |

| **Descriptive Statistics** | | | | |
| --- | --- | --- | --- | --- |
|  | Groups | Mean | Std. Deviation | N |
| MLkneeAPRE | noramweieght | -.2155 | 1.08521 | 24 |
|  | over weight | .0737 | .44472 | 24 |
|  | Total | -.0709 | .83334 | 48 |
| MLkneeAPOS | noramweieght | -.1440 | .74890 | 24 |
|  | over weight | -.3046 | .75538 | 24 |
|  | Total | -.2243 | .74851 | 48 |

| **Multivariate Tests^a^** | | | | | | | | | |
| --- | --- | --- | --- | --- | --- | --- | --- | --- | --- |
| Effect | | Value | F | Hypothesis df | Error df | Sig. | Partial Eta Squared | Noncent. Parameter | Observed Power^c^ |
| factor1 | Pillai's Trace | .038 | 1.828^b^ | 1.000 | 46.000 | .183 | .038 | 1.828 | .263 |
|  | Wilks' Lambda | .962 | 1.828^b^ | 1.000 | 46.000 | .183 | .038 | 1.828 | .263 |
|  | Hotelling's Trace | .040 | 1.828^b^ | 1.000 | 46.000 | .183 | .038 | 1.828 | .263 |
|  | Roy's Largest Root | .040 | 1.828^b^ | 1.000 | 46.000 | .183 | .038 | 1.828 | .263 |
| factor1 * Groups | Pillai's Trace | .079 | 3.929^b^ | 1.000 | 46.000 | .053 | .079 | 3.929 | .492 |
|  | Wilks' Lambda | .921 | 3.929^b^ | 1.000 | 46.000 | .053 | .079 | 3.929 | .492 |
|  | Hotelling's Trace | .085 | 3.929^b^ | 1.000 | 46.000 | .053 | .079 | 3.929 | .492 |
|  | Roy's Largest Root | .085 | 3.929^b^ | 1.000 | 46.000 | .053 | .079 | 3.929 | .492 |
| a. Design: Intercept + Groups  Within Subjects Design: factor1 | | | | | | | | | |
| b. Exact statistic | | | | | | | | | |
| c. Computed using alpha = .05 | | | | | | | | | |

| **Tests of Within-Subjects Effects** | | | | | | | | | |
| --- | --- | --- | --- | --- | --- | --- | --- | --- | --- |
| Measure: MEASURE_1 | | | | | | | | | |
| Source | | Type III Sum of Squares | df | Mean Square | F | Sig. | Partial Eta Squared | Noncent. Parameter | Observed Power^a^ |
| factor1 | Sphericity Assumed | .565 | 1 | .565 | 1.828 | .183 | .038 | 1.828 | .263 |
|  | Greenhouse-Geisser | .565 | 1.000 | .565 | 1.828 | .183 | .038 | 1.828 | .263 |
|  | Huynh-Feldt | .565 | 1.000 | .565 | 1.828 | .183 | .038 | 1.828 | .263 |
|  | Lower-bound | .565 | 1.000 | .565 | 1.828 | .183 | .038 | 1.828 | .263 |
| factor1 * Groups | Sphericity Assumed | 1.214 | 1 | 1.214 | 3.929 | .053 | .079 | 3.929 | .492 |
|  | Greenhouse-Geisser | 1.214 | 1.000 | 1.214 | 3.929 | .053 | .079 | 3.929 | .492 |
|  | Huynh-Feldt | 1.214 | 1.000 | 1.214 | 3.929 | .053 | .079 | 3.929 | .492 |
|  | Lower-bound | 1.214 | 1.000 | 1.214 | 3.929 | .053 | .079 | 3.929 | .492 |
| Error(factor1) | Sphericity Assumed | 14.208 | 46 | .309 |  |  |  |  |  |
|  | Greenhouse-Geisser | 14.208 | 46.000 | .309 |  |  |  |  |  |
|  | Huynh-Feldt | 14.208 | 46.000 | .309 |  |  |  |  |  |
|  | Lower-bound | 14.208 | 46.000 | .309 |  |  |  |  |  |
| a. Computed using alpha = .05 | | | | | | | | | |

| **Tests of Within-Subjects Contrasts** | | | | | | | | | |
| --- | --- | --- | --- | --- | --- | --- | --- | --- | --- |
| Measure: MEASURE_1 | | | | | | | | | |
| Source | factor1 | Type III Sum of Squares | df | Mean Square | F | Sig. | Partial Eta Squared | Noncent. Parameter | Observed Power^a^ |
| factor1 | Linear | .565 | 1 | .565 | 1.828 | .183 | .038 | 1.828 | .263 |
| factor1 * Groups | Linear | 1.214 | 1 | 1.214 | 3.929 | .053 | .079 | 3.929 | .492 |
| Error(factor1) | Linear | 14.208 | 46 | .309 |  |  |  |  |  |
| a. Computed using alpha = .05 | | | | | | | | | |

| **Tests of Between-Subjects Effects** | | | | | | | | |
| --- | --- | --- | --- | --- | --- | --- | --- | --- |
| Measure: MEASURE_1 | | | | | | | | |
| Transformed Variable: Average | | | | | | | | |
| Source | Type III Sum of Squares | df | Mean Square | F | Sig. | Partial Eta Squared | Noncent. Parameter | Observed Power^a^ |
| Intercept | 2.092 | 1 | 2.092 | 2.214 | .144 | .046 | 2.214 | .308 |
| Groups | .099 | 1 | .099 | .105 | .747 | .002 | .105 | .062 |
| Error | 43.451 | 46 | .945 |  |  |  |  |  |
| a. Computed using alpha = .05 | | | | | | | | |

| **Parameter Estimates** | | | | | | | | | | |
| --- | --- | --- | --- | --- | --- | --- | --- | --- | --- | --- |
| Dependent Variable | Parameter | B | Std. Error | t | Sig. | 95% Confidence Interval | | Partial Eta Squared | Noncent. Parameter | Observed Power^b^ |
|  |  |  |  |  |  | Lower Bound | Upper Bound |  |  |  |
| MLkneeAPRE | Intercept | .074 | .169 | .435 | .665 | -.267 | .414 | .004 | .435 | .071 |
|  | [Groups=1.00] | -.289 | .239 | -1.208 | .233 | -.771 | .193 | .031 | 1.208 | .219 |
|  | [Groups=2.00] | 0^a^ | . | . | . | . | . | . | . | . |
| MLkneeAPOS | Intercept | -.305 | .154 | -1.984 | .053 | -.614 | .004 | .079 | 1.984 | .493 |
|  | [Groups=1.00] | .161 | .217 | .739 | .463 | -.277 | .598 | .012 | .739 | .112 |
|  | [Groups=2.00] | 0^a^ | . | . | . | . | . | . | . | . |
| a. This parameter is set to zero because it is redundant. | | | | | | | | | | |
| b. Computed using alpha = .05 | | | | | | | | | | |

**General co-contraction knee- Late stance**

| **Within-Subjects Factors** | |
| --- | --- |
| Measure: MEASURE_1 | |
| factor1 | Dependent Variable |
| 1 | generalCoKneePreB |
| 2 | generalCoKneePosB |

| **Between-Subjects Factors** | | | |
| --- | --- | --- | --- |
|  | | Value Label | N |
| Groups | 1.00 | noramweieght | 24 |
|  | 2.00 | over weight | 24 |

| **Descriptive Statistics** | | | | |
| --- | --- | --- | --- | --- |
|  | Groups | Mean | Std. Deviation | N |
| generalCoKneePreB | noramweieght | 267.4210 | 98.20973 | 24 |
|  | over weight | 278.6244 | 91.97823 | 24 |
|  | Total | 273.0227 | 94.29746 | 48 |
| generalCoKneePosB | noramweieght | 362.3959 | 129.86017 | 24 |
|  | over weight | 417.5359 | 186.50157 | 24 |
|  | Total | 389.9659 | 161.40042 | 48 |

| **Multivariate Tests^a^** | | | | | | | | | |
| --- | --- | --- | --- | --- | --- | --- | --- | --- | --- |
| Effect | | Value | F | Hypothesis df | Error df | Sig. | Partial Eta Squared | Noncent. Parameter | Observed Power^c^ |
| factor1 | Pillai's Trace | .400 | 30.672^b^ | 1.000 | 46.000 | .000 | .400 | 30.672 | 1.000 |
|  | Wilks' Lambda | .600 | 30.672^b^ | 1.000 | 46.000 | .000 | .400 | 30.672 | 1.000 |
|  | Hotelling's Trace | .667 | 30.672^b^ | 1.000 | 46.000 | .000 | .400 | 30.672 | 1.000 |
|  | Roy's Largest Root | .667 | 30.672^b^ | 1.000 | 46.000 | .000 | .400 | 30.672 | 1.000 |
| factor1 * Groups | Pillai's Trace | .023 | 1.082^b^ | 1.000 | 46.000 | .304 | .023 | 1.082 | .175 |
|  | Wilks' Lambda | .977 | 1.082^b^ | 1.000 | 46.000 | .304 | .023 | 1.082 | .175 |
|  | Hotelling's Trace | .024 | 1.082^b^ | 1.000 | 46.000 | .304 | .023 | 1.082 | .175 |
|  | Roy's Largest Root | .024 | 1.082^b^ | 1.000 | 46.000 | .304 | .023 | 1.082 | .175 |
| a. Design: Intercept + Groups  Within Subjects Design: factor1 | | | | | | | | | |
| b. Exact statistic | | | | | | | | | |
| c. Computed using alpha = .05 | | | | | | | | | |

| **Tests of Within-Subjects Effects** | | | | | | | | | |
| --- | --- | --- | --- | --- | --- | --- | --- | --- | --- |
| Measure: MEASURE_1 | | | | | | | | | |
| Source | | Type III Sum of Squares | df | Mean Square | F | Sig. | Partial Eta Squared | Noncent. Parameter | Observed Power^a^ |
| factor1 | Sphericity Assumed | 328216.884 | 1 | 328216.884 | 30.672 | .000 | .400 | 30.672 | 1.000 |
|  | Greenhouse-Geisser | 328216.884 | 1.000 | 328216.884 | 30.672 | .000 | .400 | 30.672 | 1.000 |
|  | Huynh-Feldt | 328216.884 | 1.000 | 328216.884 | 30.672 | .000 | .400 | 30.672 | 1.000 |
|  | Lower-bound | 328216.884 | 1.000 | 328216.884 | 30.672 | .000 | .400 | 30.672 | 1.000 |
| factor1 * Groups | Sphericity Assumed | 11582.531 | 1 | 11582.531 | 1.082 | .304 | .023 | 1.082 | .175 |
|  | Greenhouse-Geisser | 11582.531 | 1.000 | 11582.531 | 1.082 | .304 | .023 | 1.082 | .175 |
|  | Huynh-Feldt | 11582.531 | 1.000 | 11582.531 | 1.082 | .304 | .023 | 1.082 | .175 |
|  | Lower-bound | 11582.531 | 1.000 | 11582.531 | 1.082 | .304 | .023 | 1.082 | .175 |
| Error(factor1) | Sphericity Assumed | 492241.829 | 46 | 10700.909 |  |  |  |  |  |
|  | Greenhouse-Geisser | 492241.829 | 46.000 | 10700.909 |  |  |  |  |  |
|  | Huynh-Feldt | 492241.829 | 46.000 | 10700.909 |  |  |  |  |  |
|  | Lower-bound | 492241.829 | 46.000 | 10700.909 |  |  |  |  |  |
| a. Computed using alpha = .05 | | | | | | | | | |

| **Tests of Within-Subjects Contrasts** | | | | | | | | | |
| --- | --- | --- | --- | --- | --- | --- | --- | --- | --- |
| Measure: MEASURE_1 | | | | | | | | | |
| Source | factor1 | Type III Sum of Squares | df | Mean Square | F | Sig. | Partial Eta Squared | Noncent. Parameter | Observed Power^a^ |
| factor1 | Linear | 328216.884 | 1 | 328216.884 | 30.672 | .000 | .400 | 30.672 | 1.000 |
| factor1 * Groups | Linear | 11582.531 | 1 | 11582.531 | 1.082 | .304 | .023 | 1.082 | .175 |
| Error(factor1) | Linear | 492241.829 | 46 | 10700.909 |  |  |  |  |  |
| a. Computed using alpha = .05 | | | | | | | | | |

| **Tests of Between-Subjects Effects** | | | | | | | | |
| --- | --- | --- | --- | --- | --- | --- | --- | --- |
| Measure: MEASURE_1 | | | | | | | | |
| Transformed Variable: Average | | | | | | | | |
| Source | Type III Sum of Squares | df | Mean Square | F | Sig. | Partial Eta Squared | Noncent. Parameter | Observed Power^a^ |
| Intercept | 10549293.603 | 1 | 10549293.603 | 436.374 | .000 | .905 | 436.374 | 1.000 |
| Groups | 26408.654 | 1 | 26408.654 | 1.092 | .301 | .023 | 1.092 | .176 |
| Error | 1112045.981 | 46 | 24174.913 |  |  |  |  |  |
| a. Computed using alpha = .05 | | | | | | | | |

| **Parameter Estimates** | | | | | | | | | | |
| --- | --- | --- | --- | --- | --- | --- | --- | --- | --- | --- |
| Dependent Variable | Parameter | B | Std. Error | t | Sig. | 95% Confidence Interval | | Partial Eta Squared | Noncent. Parameter | Observed Power^b^ |
|  |  |  |  |  |  | Lower Bound | Upper Bound |  |  |  |
| generalCoKneePreB | Intercept | 278.624 | 19.421 | 14.346 | .000 | 239.531 | 317.718 | .817 | 14.346 | 1.000 |
|  | [Groups=1.00] | -11.203 | 27.466 | -.408 | .685 | -66.490 | 44.083 | .004 | .408 | .068 |
|  | [Groups=2.00] | 0^a^ | . | . | . | . | . | . | . | . |
| generalCoKneePosB | Intercept | 417.536 | 32.802 | 12.729 | .000 | 351.509 | 483.563 | .779 | 12.729 | 1.000 |
|  | [Groups=1.00] | -55.140 | 46.389 | -1.189 | .241 | -148.516 | 38.236 | .030 | 1.189 | .214 |
|  | [Groups=2.00] | 0^a^ | . | . | . | . | . | . | . | . |
| a. This parameter is set to zero because it is redundant. | | | | | | | | | | |
| b. Computed using alpha = .05 | | | | | | | | | | |

**Directed**

**knee Flexor/Extensor co-contraction – Late stance**

| **Within-Subjects Factors** | |
| --- | --- |
| Measure: MEASURE_1 | |
| factor1 | Dependent Variable |
| 1 | flexexkneeBPRE |
| 2 | flexexkneeBPOS |

| **Between-Subjects Factors** | | | |
| --- | --- | --- | --- |
|  | | Value Label | N |
| Groups | 1.00 | noramweieght | 24 |
|  | 2.00 | over weight | 24 |

| **Descriptive Statistics** | | | | |
| --- | --- | --- | --- | --- |
|  | Groups | Mean | Std. Deviation | N |
| flexexkneeBPRE | noramweieght | .4591 | .32981 | 24 |
|  | over weight | .3616 | .46478 | 24 |
|  | Total | .4103 | .40171 | 48 |
| flexexkneeBPOS | noramweieght | .3546 | .72562 | 24 |
|  | over weight | .5530 | .23609 | 24 |
|  | Total | .4538 | .54312 | 48 |

| **Multivariate Tests^a^** | | | | | | | | | |
| --- | --- | --- | --- | --- | --- | --- | --- | --- | --- |
| Effect | | Value | F | Hypothesis df | Error df | Sig. | Partial Eta Squared | Noncent. Parameter | Observed Power^c^ |
| factor1 | Pillai's Trace | .005 | .210^b^ | 1.000 | 46.000 | .649 | .005 | .210 | .073 |
|  | Wilks' Lambda | .995 | .210^b^ | 1.000 | 46.000 | .649 | .005 | .210 | .073 |
|  | Hotelling's Trace | .005 | .210^b^ | 1.000 | 46.000 | .649 | .005 | .210 | .073 |
|  | Roy's Largest Root | .005 | .210^b^ | 1.000 | 46.000 | .649 | .005 | .210 | .073 |
| factor1 * Groups | Pillai's Trace | .050 | 2.427^b^ | 1.000 | 46.000 | .126 | .050 | 2.427 | .332 |
|  | Wilks' Lambda | .950 | 2.427^b^ | 1.000 | 46.000 | .126 | .050 | 2.427 | .332 |
|  | Hotelling's Trace | .053 | 2.427^b^ | 1.000 | 46.000 | .126 | .050 | 2.427 | .332 |
|  | Roy's Largest Root | .053 | 2.427^b^ | 1.000 | 46.000 | .126 | .050 | 2.427 | .332 |
| a. Design: Intercept + Groups  Within Subjects Design: factor1 | | | | | | | | | |
| b. Exact statistic | | | | | | | | | |
| c. Computed using alpha = .05 | | | | | | | | | |

| **Tests of Within-Subjects Effects** | | | | | | | | | |
| --- | --- | --- | --- | --- | --- | --- | --- | --- | --- |
| Measure: MEASURE_1 | | | | | | | | | |
| Source | | Type III Sum of Squares | df | Mean Square | F | Sig. | Partial Eta Squared | Noncent. Parameter | Observed Power^a^ |
| factor1 | Sphericity Assumed | .045 | 1 | .045 | .210 | .649 | .005 | .210 | .073 |
|  | Greenhouse-Geisser | .045 | 1.000 | .045 | .210 | .649 | .005 | .210 | .073 |
|  | Huynh-Feldt | .045 | 1.000 | .045 | .210 | .649 | .005 | .210 | .073 |
|  | Lower-bound | .045 | 1.000 | .045 | .210 | .649 | .005 | .210 | .073 |
| factor1 * Groups | Sphericity Assumed | .525 | 1 | .525 | 2.427 | .126 | .050 | 2.427 | .332 |
|  | Greenhouse-Geisser | .525 | 1.000 | .525 | 2.427 | .126 | .050 | 2.427 | .332 |
|  | Huynh-Feldt | .525 | 1.000 | .525 | 2.427 | .126 | .050 | 2.427 | .332 |
|  | Lower-bound | .525 | 1.000 | .525 | 2.427 | .126 | .050 | 2.427 | .332 |
| Error(factor1) | Sphericity Assumed | 9.959 | 46 | .217 |  |  |  |  |  |
|  | Greenhouse-Geisser | 9.959 | 46.000 | .217 |  |  |  |  |  |
|  | Huynh-Feldt | 9.959 | 46.000 | .217 |  |  |  |  |  |
|  | Lower-bound | 9.959 | 46.000 | .217 |  |  |  |  |  |
| a. Computed using alpha = .05 | | | | | | | | | |

| **Tests of Within-Subjects Contrasts** | | | | | | | | | |
| --- | --- | --- | --- | --- | --- | --- | --- | --- | --- |
| Measure: MEASURE_1 | | | | | | | | | |
| Source | factor1 | Type III Sum of Squares | df | Mean Square | F | Sig. | Partial Eta Squared | Noncent. Parameter | Observed Power^a^ |
| factor1 | Linear | .045 | 1 | .045 | .210 | .649 | .005 | .210 | .073 |
| factor1 * Groups | Linear | .525 | 1 | .525 | 2.427 | .126 | .050 | 2.427 | .332 |
| Error(factor1) | Linear | 9.959 | 46 | .217 |  |  |  |  |  |
| a. Computed using alpha = .05 | | | | | | | | | |

| **Tests of Between-Subjects Effects** | | | | | | | | |
| --- | --- | --- | --- | --- | --- | --- | --- | --- |
| Measure: MEASURE_1 | | | | | | | | |
| Transformed Variable: Average | | | | | | | | |
| Source | Type III Sum of Squares | df | Mean Square | F | Sig. | Partial Eta Squared | Noncent. Parameter | Observed Power^a^ |
| Intercept | 17.922 | 1 | 17.922 | 75.610 | .000 | .622 | 75.610 | 1.000 |
| Groups | .061 | 1 | .061 | .258 | .614 | .006 | .258 | .079 |
| Error | 10.903 | 46 | .237 |  |  |  |  |  |
| a. Computed using alpha = .05 | | | | | | | | |

| **Parameter Estimates** | | | | | | | | | | |
| --- | --- | --- | --- | --- | --- | --- | --- | --- | --- | --- |
| Dependent Variable | Parameter | B | Std. Error | t | Sig. | 95% Confidence Interval | | Partial Eta Squared | Noncent. Parameter | Observed Power^b^ |
|  |  |  |  |  |  | Lower Bound | Upper Bound |  |  |  |
| flexexkneeBPRE | Intercept | .362 | .082 | 4.395 | .000 | .196 | .527 | .296 | 4.395 | .990 |
|  | [Groups=1.00] | .098 | .116 | .838 | .406 | -.137 | .332 | .015 | .838 | .130 |
|  | [Groups=2.00] | 0^a^ | . | . | . | . | . | . | . | . |
| flexexkneeBPOS | Intercept | .553 | .110 | 5.021 | .000 | .331 | .775 | .354 | 5.021 | .998 |
|  | [Groups=1.00] | -.198 | .156 | -1.274 | .209 | -.512 | .115 | .034 | 1.274 | .239 |
|  | [Groups=2.00] | 0^a^ | . | . | . | . | . | . | . | . |
| a. This parameter is set to zero because it is redundant. | | | | | | | | | | |
| b. Computed using alpha = .05 | | | | | | | | | | |

**Directed knee medio/lateral co-contraction-Late stance**

| **Within-Subjects Factors** | |
| --- | --- |
| Measure: MEASURE_1 | |
| factor1 | Dependent Variable |
| 1 | MLkneeBPRE |
| 2 | MLkneeBPOS |

| **Between-Subjects Factors** | | | |
| --- | --- | --- | --- |
|  | | Value Label | N |
| Groups | 1.00 | noramweieght | 24 |
|  | 2.00 | over weight | 24 |

| **Descriptive Statistics** | | | | |
| --- | --- | --- | --- | --- |
|  | Groups | Mean | Std. Deviation | N |
| MLkneeBPRE | noramweieght | .1409 | .45248 | 24 |
|  | over weight | .1730 | .25332 | 24 |
|  | Total | .1570 | .36312 | 48 |
| MLkneeBPOS | noramweieght | -.2188 | .56585 | 24 |
|  | over weight | -.2122 | .81674 | 24 |
|  | Total | -.2155 | .69508 | 48 |

| **Multivariate Tests^a^** | | | | | | | | | |
| --- | --- | --- | --- | --- | --- | --- | --- | --- | --- |
| Effect | | Value | F | Hypothesis df | Error df | Sig. | Partial Eta Squared | Noncent. Parameter | Observed Power^c^ |
| factor1 | Pillai's Trace | .241 | 14.577^b^ | 1.000 | 46.000 | .000 | .241 | 14.577 | .962 |
|  | Wilks' Lambda | .759 | 14.577^b^ | 1.000 | 46.000 | .000 | .241 | 14.577 | .962 |
|  | Hotelling's Trace | .317 | 14.577^b^ | 1.000 | 46.000 | .000 | .241 | 14.577 | .962 |
|  | Roy's Largest Root | .317 | 14.577^b^ | 1.000 | 46.000 | .000 | .241 | 14.577 | .962 |
| factor1 * Groups | Pillai's Trace | .000 | .017^b^ | 1.000 | 46.000 | .896 | .000 | .017 | .052 |
|  | Wilks' Lambda | 1.000 | .017^b^ | 1.000 | 46.000 | .896 | .000 | .017 | .052 |
|  | Hotelling's Trace | .000 | .017^b^ | 1.000 | 46.000 | .896 | .000 | .017 | .052 |
|  | Roy's Largest Root | .000 | .017^b^ | 1.000 | 46.000 | .896 | .000 | .017 | .052 |
| a. Design: Intercept + Groups  Within Subjects Design: factor1 | | | | | | | | | |
| b. Exact statistic | | | | | | | | | |
| c. Computed using alpha = .05 | | | | | | | | | |

| **Tests of Within-Subjects Effects** | | | | | | | | | |
| --- | --- | --- | --- | --- | --- | --- | --- | --- | --- |
| Measure: MEASURE_1 | | | | | | | | | |
| Source | | Type III Sum of Squares | df | Mean Square | F | Sig. | Partial Eta Squared | Noncent. Parameter | Observed Power^a^ |
| factor1 | Sphericity Assumed | 3.329 | 1 | 3.329 | 14.577 | .000 | .241 | 14.577 | .962 |
|  | Greenhouse-Geisser | 3.329 | 1.000 | 3.329 | 14.577 | .000 | .241 | 14.577 | .962 |
|  | Huynh-Feldt | 3.329 | 1.000 | 3.329 | 14.577 | .000 | .241 | 14.577 | .962 |
|  | Lower-bound | 3.329 | 1.000 | 3.329 | 14.577 | .000 | .241 | 14.577 | .962 |
| factor1 * Groups | Sphericity Assumed | .004 | 1 | .004 | .017 | .896 | .000 | .017 | .052 |
|  | Greenhouse-Geisser | .004 | 1.000 | .004 | .017 | .896 | .000 | .017 | .052 |
|  | Huynh-Feldt | .004 | 1.000 | .004 | .017 | .896 | .000 | .017 | .052 |
|  | Lower-bound | .004 | 1.000 | .004 | .017 | .896 | .000 | .017 | .052 |
| Error(factor1) | Sphericity Assumed | 10.506 | 46 | .228 |  |  |  |  |  |
|  | Greenhouse-Geisser | 10.506 | 46.000 | .228 |  |  |  |  |  |
|  | Huynh-Feldt | 10.506 | 46.000 | .228 |  |  |  |  |  |
|  | Lower-bound | 10.506 | 46.000 | .228 |  |  |  |  |  |
| a. Computed using alpha = .05 | | | | | | | | | |

| **Tests of Within-Subjects Contrasts** | | | | | | | | | |
| --- | --- | --- | --- | --- | --- | --- | --- | --- | --- |
| Measure: MEASURE_1 | | | | | | | | | |
| Source | factor1 | Type III Sum of Squares | df | Mean Square | F | Sig. | Partial Eta Squared | Noncent. Parameter | Observed Power^a^ |
| factor1 | Linear | 3.329 | 1 | 3.329 | 14.577 | .000 | .241 | 14.577 | .962 |
| factor1 * Groups | Linear | .004 | 1 | .004 | .017 | .896 | .000 | .017 | .052 |
| Error(factor1) | Linear | 10.506 | 46 | .228 |  |  |  |  |  |
| a. Computed using alpha = .05 | | | | | | | | | |

|  |
| --- |

| **Tests of Between-Subjects Effects** | | | | | | | | |
| --- | --- | --- | --- | --- | --- | --- | --- | --- |
| Measure: MEASURE_1 | | | | | | | | |
| Transformed Variable: Average | | | | | | | | |
| Source | Type III Sum of Squares | df | Mean Square | F | Sig. | Partial Eta Squared | Noncent. Parameter | Observed Power^a^ |
| Intercept | .082 | 1 | .082 | .206 | .652 | .004 | .206 | .073 |
| Groups | .009 | 1 | .009 | .023 | .881 | .000 | .023 | .052 |
| Error | 18.386 | 46 | .400 |  |  |  |  |  |
| a. Computed using alpha = .05 | | | | | | | | |

| **Parameter Estimates** | | | | | | | | | | |
| --- | --- | --- | --- | --- | --- | --- | --- | --- | --- | --- |
| Dependent Variable | Parameter | B | Std. Error | t | Sig. | 95% Confidence Interval | | Partial Eta Squared | Noncent. Parameter | Observed Power^b^ |
|  |  |  |  |  |  | Lower Bound | Upper Bound |  |  |  |
| MLkneeBPRE | Intercept | .173 | .075 | 2.312 | .025 | .022 | .324 | .104 | 2.312 | .619 |
|  | [Groups=1.00] | -.032 | .106 | -.304 | .763 | -.245 | .181 | .002 | .304 | .060 |
|  | [Groups=2.00] | 0^a^ | . | . | . | . | . | . | . | . |
| MLkneeBPOS | Intercept | -.212 | .143 | -1.479 | .146 | -.501 | .077 | .045 | 1.479 | .305 |
|  | [Groups=1.00] | -.007 | .203 | -.033 | .974 | -.415 | .402 | .000 | .033 | .050 |
|  | [Groups=2.00] | 0^a^ | . | . | . | . | . | . | . | . |
| a. This parameter is set to zero because it is redundant. | | | | | | | | | | |
| b. Computed using alpha = .05 | | | | | | | | | | |
